# Using landscape genomics to infer genomic regions involved in environmental adaptation of soybean genebank accessions

**DOI:** 10.1186/s12870-025-07202-5

**Published:** 2025-09-01

**Authors:** Max Haupt, Karl Schmid

**Affiliations:** 1 Institute of Plant Breeding, Seed Science and Population Genetics, University of Hohenheim, Stuttgart, Germany; 2 Leibniz Institute of Plant Genetics and Crop Plant Research (IPK), Seeland OT Gatersleben, Germany

**Keywords:** *Glycine max*, Environmental association mapping, Local adaptation, Landscape genomics, Population structure, Genetic differentiation, Genetic resources, Genebank accessions

## Abstract

**Background:**

Understanding how crops adapt to specific environmental conditions is becoming increasingly important in the face of accelerating climate change, but the genetics of local adaptation remains little understood for many crops. Landscape genomics can reveal patterns of genetic variation that indicate adaptive diversification during crop evolution and dispersal. Here, we examine genetic differentiation and association signatures with environmental gradients in soybean (*Glycine max*) germplasm groups from China that were inferred from the USDA Soybean Germplasm Collection (*N* = 17, 019 accessions) based on population structure and passport information.

**Results:**

We recover genes previously known to be involved in soybean environmental adaptation and report numerous new candidate genes in adaptation signatures implicated by genomic resources such as the genome annotation and gene expression datasets to function in flowering regulation, photoperiodism and stress reaction cascades. Linkage disequilibrium network analysis suggested functional relationships between genomic regions with signatures of genetic differentiation, consistent with a polygenic nature of environmental adaptation. We tested whether haplotypes associated with environmental adaptation in China were present in 843 North American and 160 European soybean cultivars and found that haplotypes in major genes for early maturity have been selected during breeding, but also that a large number of haplotypes exhibiting putative adaptive variation for cold regions at high latitudes are underrepresented in modern cultivars.

**Conclusions:**

Our results demonstrate the value of landscape genomics analysis of genebank accessions studying crop environmental adaptation and to inform future research and breeding efforts for improved adaptation of soybean and other crops to future climates.

**Supplementary Information:**

The online version contains supplementary material available at 10.1186/s12870-025-07202-5.

## Background

The environmental adaptation of crop varieties is of central importance in plant breeding and a key objective for successful crop production. Modern plant breeding addresses this need by classifying target production environments into homogeneous mega-environments, which are defined by the spatial distribution of both biotic and abiotic stresses [1]. This approach contributed to the efficient development of crop varieties with region-specific adaptation. Increasing environmental variability caused by ongoing climate change results in more frequent and severe environmental stresses [2], demanding the breeding of more resilient varieties that maintain high yield levels. In addition, a precise adaptation of cultivars to production environments at finer resolution than current mega-environments may increase yields per unit of agricultural land [3]. These objectives require a detailed understanding of the genetic architecture of environmental adaptation and stress resistance to develop highly adapted varieties for specific target production environments, which is challenging because of the highly polygenic nature of adaptive traits [4]. In plant breeding, functional studies of adaptation are frequently limited to stress experiments conducted under controlled conditions in which few stress factors and their interactions are evaluated with a small number of genotypes. Breeding of new, climate-resistant varieties requires the identification of new sources of beneficial genetic variation for polygenic traits by screening large sets of genetic resources, and a detailed characterisation of the genetic architecture of adaptation.

Landscape genomics investigates the genetic basis of environmental adaptation, using evidence from natural experiments that influenced genetic diversity during plant evolution. This method identifies signs of natural selection in the genome, revealing mechanisms of local adaptation [5]. Key approaches include outlier tests, which identify allele frequency differences between populations, and genotype-environment association (GEA) tests. These GEA tests uncover correlations between environmental variables and allele frequencies in genomic regions linked to local adaptation [6]. The diversification and spatial expansion of crop species after their domestication has resulted in locally adapted cultivars, influenced by both natural and human-mediated selection [7]. Landscape genomics can examine adaptive diversification among these cultivars, finding valuable genetic variation for contemporary breeding. It can therefore effectively utilize germplasm from ex situ genebanks and contribute to the breeding of future-ready crops.

After being domesticated in China approximately 3,000 years ago, the cultivation range of soybean (*Glycine max*) has significantly expanded beyond its original center of domestication. This expansion gave rise to over 20,000 landraces [8]. By the 15th century AD, soybean cultivation spread across Asia, and by the 1930 s, it had become a prominent crop in the Americas. Today, it is one of the most economically significant crops worldwide [9]. Adaptation to variable day lengths across different latitudes was crucial for its expansion and the *E*-series loci in soybean were identified as the primary genes controlling flowering time and maturity by regulating its response to day length, i.e., the photoperiod [10]. Notably, genetic variation at the *E1 - E4* loci have been documented in multiple germplasm sets [reviewed by 11]. The geographic distribution of genetic diversity at these loci suggest regional adaptation [12, 13]. Current studies of soybean adaptation, based on phenotypic evaluation, often validate the alignment of allelic variation at these genes with environmental gradients [14–16]. Hence, these loci are prime candidates for genome scans using landscape genomics methods. Further research on both soybean and its wild relative, *G. soja*, revealed putative adaptive correlations with environmental gradients in East Asia, including humidity, precipitation, and temperature. Such gradients potentially affect traits such as pod dehiscence [17], root growth, and drought resilience [18, 19].

In this study, we expand previous work on soybean adaptation [20] by focusing on differences in genetic variation among germplasm groups native to China. We also explore associations of population allele frequencies with environmental gradients to identify the genetic basis of local environmental adaptation. By combining passport data of the global USDA Soybean Germplasm Collection with the inferred population structure of this material we identified subpopulations and assigned them to their original cultivation environments. Using the population and environmental classification, we carried out landscape genomic analyses to pinpoint genomic regions exhibiting adaptive differences between germplasm groups cultivated in different environments. Through analyses of linkage disequilibrium, genome annotation, and gene expression data, we elucidated the genomic architecture of adaptation and identified putative functions of candidate genes located in regions showing adaptation signatures. Finally, we determined which putative adaptive haplotypes are present in U.S. and European varieties. Our results demonstrate the value of landscape genomic analysis of genebank germplasm for studying crop environmental adaptation and its potential to aid soybean adaptation to new environmental conditions.

## Methods

### Plant materials

The soybean material analyzed in this study primarily originates from the USDA Soybean Germplasm Collection [21]. This collection spans maturity groups (MG) from 000 (early) to MG X (late), representing a global germplasm sample (*N* = 17,019) with a focus on origin from Asia. Additionally, the dataset encompasses old U.S. cultivars (*N* = 208; released roughly between 1895 and the 1940 s) and a selection of modern cultivars from both the U.S. and Canada, originating from public and private breeding programs (*N* = 635; released approximately between 1947 and 2016). We also incorporated 160 contemporary European varieties from maturity groups 000 to II, reflecting the spectrum of soybean germplasm currently cultivated in Europe.

### Genotyping data

We obtained the SoySNP50K SNP array genotyping data of the USDA Soybean Germplasm Collection from SoyBase (https://soybase.org/snps/; [22, 23]). This dataset comprises 42,080 markers with an average percentage of less than 1% missing data, both per individual and per marker. A set of 160 modern European varieties was genotyped using the same array on an Illumina iScan system at TraitGenetics, Germany. DNA was extracted from the dried leaf tissue of a single plant from each variety using the Genomic Micro AX Blood Gravity Kit from A&A Biotechnology. We successfully genotyped 48,718 SNP variants in European varieties with an average missing data percentage below 1%. We merged the USDA and our genotyping data into a single VCF file using VCFtools [24]. SNPs without a chromosomal location and markers that were only polymorphic in the 160 European varieties were excluded. We imputed any remaining missing data using BEAGLE version 5.1 with default settings [25]. To assess imputation accuracy, we masked a random subset (10%) of the marker dataset before imputation. The finalized SoySNP50K dataset comprised 20,269 accessions with genotype data for 41,084 SNP markers and achieved an imputation accuracy of 98.30%. A total of 17,362 accessions exhibited heterozygosity levels below 1%, whereas only four accessions exceeded 20% heterozygosity. Similarly, heterozygosity among markers was low: 80% of markers showed up to 1% heterozygosity across all accessions. Only 58 markers displayed heterozygosity above 5%.

We obtained high-density SNP genotypes for accessions from the USDA Soybean Germplasm Collection, derived from the soybean haplotype map (GmHapMap). This resource is built on an imputation from SoySNP50K genotypes and resequencing data [26]. From the GmHapMap, we extracted genotypes for 1,508 accessions that our population structure analysis categorized into one of four Chinese subpopulations. Using VCFtools [24], we retained 1,088,360 SNP markers with a minor allele frequency *≥* 1%. This dataset assisted in identifying adaptation signatures in genebank accessions originating from China. We assembled a second dataset encompassing the same SNP markers for varieties in the GmHapMap resource from the USA and Canada. This dataset facilitated a comparative analysis with the Chinese germplasm to identify genomic regions displaying adaptation signatures. To enable a comparison with modern European cultivars, we combined the SoySNP50K genotypes for 160 European cultivars with both the SoySNP50K and GmHapMap genotypes for the 1,508 Chinese accessions. We used BEAGLE version 5.1 [25] to impute missing GmHapMap markers for these 160 cultivars, achieving an imputation accuracy of 98.55%. Finally, we extracted the GmHapMap genotypes for the 160 European cultivars from this compiled dataset.

### Inference of population structure

We utilized ADMIXTURE v1.23 [27] to estimate ancestry in the global germplasm sample, using the. SoySNP50K genotypes for markers with a minor allele frequency of *≥* 0.05 (*N* = 34,358 SNPs). Since ADMIXTURE assumes linkage equilibrium between markers, we pruned the data by excluding markers that exceeded background LD levels in sliding windows of 500 kb and included 10 markers. This pruning was conducted with the snpgdsLDpruning function from the SNPRelate R package [28], and produced a refined marker set of 5,283 SNPs. For inference of population structure, we conducted ten independent ADMIXTURE runs for various model scenarios, each differing in the presumed number of ancestral populations (*K*). We assessed models ranging from *K* = 2 to *K* = 20, leveraging 10-fold cross-validation to determine model accuracy. The results from these runs were aligned and summarized for each *K* using CLUMPP v1.1.2 [29]. Further, we inferred population structure by conducting a principal component analysis (PCA) on the complete SoySNP50K dataset with the dudi.pca function from the ade4 R package [30].

### Inference of environmental association and detection of adaptation signatures

**Fig. 1:**
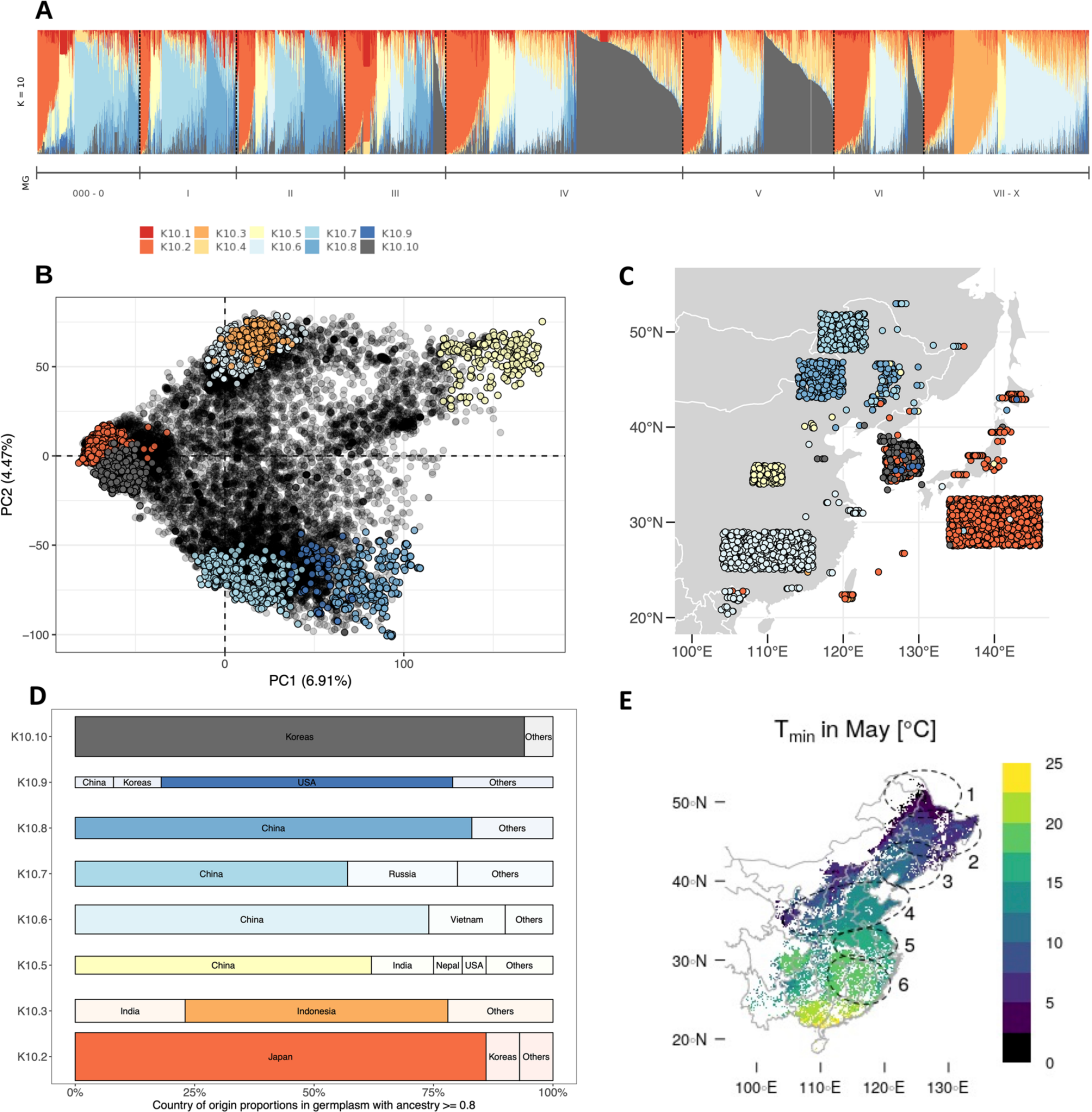
Population Structure and Geographic Origins of the USDA Soybean Germplasm Collection: (**A**) ADMIXTURE analysis of 17,019 genebank accessions with*K *= 10 based on 5,283 LD-pruned SNPs. Each vertical line represents a single accession, with segment lengths indicating ancestry proportions from the deduced ancestral populations. (**B**) Principal Component Analysis (PCA) of the 17,019 accessions based on the complete set of 34,358 SoySNP50K SNP genotypes. Accessions with an ancestry fraction >= 80% from one of the deduced ancestral groups (K10.1 and K10.10) are emphasized. (**C**) Geographical origins of conserved East Asian accessions based on georeferencing. For accessions without georeference details, origins are grouped and determined by country and province information. (**D**) Geographic origin of genebank accessions based on country. The bar width indicatives group size. (**E**) Geographic extent of soybean cultivation in China. Ellipses 1 - 6 represent soybean areas to which germplasm was allocated as shown in Table 1 and Figure S3. Average minimum temperatures for May in soybean cultivation areas are illustrated in Figure S1

To infer environmental adaptation we approximated traditional cultivation environments using the EARTHSTAT data, which integrate national, state, and county-level census statistics spanning 1995 to 2005. The data allowed us to detail soybean harvested areas at a resolution of approximately 10 km^2^, and to estimate the geographic distribution of soybean cultivation in China [31]. We then overlayed this dataset with monthly climate data from WorldClim V2.1 [32], which has a resolution of approximately 1 km^2^ to extract climate data specific to soybean cultivation zones identified with the EARTHSTAT data (Figs. 1E and S1). We calculated environmental variables such as *mean daily temperature*, *cumulative temperature ≥ 15 °C*, *average daily temperature range*, *precipitation amount*, and *average daily solar radiation* from the monthly WorldClim mean values. These variables described the vegetative and generative phases of soybean cultivation in six regions, along with their latitudinal mean values (Tables 1 and 2). We summarized the dataset using principal component analysis via the dudi.pca function of the ade4 R package [30]. Definitions of the geographical boundaries of historical cultivation regions and the region-specific soybean growing seasons were obtained from literature on Chinese soybean ecotypes [33–36]. The assignment of each genebank accession to one of these regions was done by using both the population structure inference (Fig. 1) and passport data from the USDA Germplasm Resources Information Network (GRIN) database (www.ars-grin.gov), in particular collection site information and maturity group designation (Figure S3).

We utilized the standard covariate model of BAYPASS [37] to identify genetically distinct regions among soybean populations from the six growing regions and to detect markers linked to population-specific covariates. The population structure analysis highlighted two unique genetic groups, both originating from northeastern China, with significantly overlapping distribution ranges. We therefore conducted separate BAYPASS runs for each group in conjunction with the other groups to discern both convergent and lineagespecific adaptation to the northeastern Chinese environment. These two analyses are referred to as scenario A and B, respectively (Table 1). For both scenarios, we employed the first two principal components from environmental characterization as population covariate inputs (see Table 2). The genotypic inputs consisted of population allele counts for 1,088,360 GmHapMap SNPs with minor allele frequencies of at least 1% (Supplementary Data Worksheets 2 to 5).

**Table 1:** Allocation of germplasm groups to growing regions in China based on fractions of ancestry *≥* 80% with one of the inferred ancestral populations and based on passport information. Regions 1–6 correspond to the growing regions given in Fig. 1E; Table 2. Subdivision of the K10.7 and K10.8 groups was based on maturity group ratings and group K10.6 was divided according to provincial origin. Two independent genome scans that differed by the included germplasm groups from North-Eastern China were performed: scenario A included group K10.7 and scenario B included group K10.8, respectively. NE: North-Eastern china, YR: yellow river valley, MLRY: middle to lower reaches of Yangtze river region, LSR: lower subtropical region, RP_R1−R8_: soybean reproductive period from begin of flowering to full maturity

Region	Germplasm	MG	*N*	Sowing Time	RPR1-R8	Scenario	
1: NE	K10.7	000–0	223	May 1	Jul 1 – Sep 30	A	
2: NE	K10.7	I	145	May 1	Jul 1 – Sep 30	A	
	K10.8	I	123	May 1	Jul 1 – Sep 30		B
3: NE	K10.7	II – III	152	May 1	Jul 1 – Sep 30	A	
	K10.8	II – III	250	May 1	Jul 1 – Sep 30		B
4: YR	K10.5	II – VII	245	May 1	Jul 1 – Sep 30	A	B
5: MLRY	K10.6	IV – VIII	318	Jun 15	Aug 1 – Sep 30	A	B
6: LSR	K10.6	IV – VIII	52	Jun 15	Aug 1 – Sep 30	A	B

**Table 2:** Environmental conditions in six soybean growing regions in China during the vegetative and reproductive phases of soybean development. Lat: latitude, T_avg_:*daily average temperature*, T_acc15_:*accumulated temperature ≥ 15°C*, DTR: *average diurnal temperature range*, Prec:*precipitation amount*, SRad: *average daily solar radiation*, PC1/PC2: position of the growing regions on the first two principal components from a summary of the environmental data by principal component analysis, ^1/2^: significant correlation with with PC1 or PC2 (p < 0.05), Pearson correlation coefficients are given in Figure S5

Vegetative Phase						Reproductive Phase						
Lat^1^	Tavg1	Tacc15^1^	DTR^1^	Prec^1^	SRad	Tavg1	Tacc15^2^	DTR^1^	Prec	SRad^1^	PC1	PC2
	[^◦^C]	[^◦^C]	[^◦^C]	[mm]	[kJ m^−2^day^−1^]	[^◦^C]	[^◦^C]	[^◦^C]	[mm]	[kJ m^−2^day^−1^]	(74.5%)	(23.1%)
1 49.55	13.92	496.48	13.71	123.15	20831.60	16.09	1152.07	12.48	324.39	16827.97	−3.88	1.76
2 46.09	16.17	647.31	11.95	128.94	20870.56	18.75	1393.13	11.31	319.65	17483.93	−2.03	0.78
3 42.49	17.89	934.92	11.64	137.17	21257.63	20.55	1785.42	11.88	372.49	18018.56	−1.35	−1.40
4 36.33	20.40	1181.04	12.08	111.98	21729.14	22.16	1971.41	11.15	349.50	18684.50	−0.17	−2.84
5 32.21	26.89	1236.98	9.63	260.16	20194.97	25.02	1526.49	9.97	229.66	18261.92	3.19	1.07
6 27.70	27.22	1252.21	8.58	255.22	20136.46	25.95	1583.04	9.10	234.48	18852.77	4.24	0.63

The *XtX* statistic, corresponding to a SNP-specific *F*_*ST*_ value, was calculated by BAYPASS during runs comparing genome-wide genetic differentiation among five to six germplasm groups. It also estimated SNP-wise associations between population allele frequencies and environmental variable gradients. The *XtX* statistic corrects for population structure by accounting for the variance-covariance structure between populations [38]. The significance of the observed *XtX* signals was assessed against pseudo-observed data sets (PODs) of 100,000 SNPs, obtained using the R function simulate.baypass [37]. For markercovariate associations, BAYPASS evaluates models both with and without associations and quantifies model support through Bayes factors (BFs) in deciban units. A BF exceeding 10 suggests “strong evidence” supporting a marker-covariate association with “very strong evidence” between 15 and 20, and “decisive evidence” when BF is above 20. We conducted five independent BAYPASS runs for each scenario. The results were consolidated, representing the mean *XtX* statistics and BFs for environmental associations with principal components across the five runs. We considered SNPs exceeding the 99% quantile of *XtX* PODs to indicate adaptive differentiation between populations, and SNPs with BFs greater than 10 to correlate with an environmental gradient. Proximate SNPs separated by less than 50 kb that exceeded these limits were grouped as a singular adaptation signal. For these identified regions, peak signals and boundary positions (extended by *±* 5 kb) were recorded.

### Linkage disequilibrium and LD network analysis

We examined Linkage Disequilibrium (LD) using SoySNP50K genotypes across various groups: the entire set of genebank accessions, *G. max* collection accessions, old US cultivars, modern North American cultivars, and European cultivars. For LD calculations, only SNPs with a minor allele frequency of *≥* 5% (equivalent to 34,358 SNPs) were considered. Pairwise LD (*r*^2^) between SNPs was calculated with PLINK [39] through the pairwiseLD function of the synbreed R package [40]. The background LD level from LD between physically unlinked markers was calculated according to Breseghello and Sorrels [41]. After identifying adaptation signatures, we also computed pairwise *r*^2^ values for ’peak’ markers within regions showing strong evidence for population differentiation or association signatures. This was done using the GmHapMap SNP data via the LD function of the gaston R package [42]. These calculations aimed to discern potential epistatic interactions between physically separated loci linked to polygenic local adaptation [43]. We further estimated LD using ten samples, each with 1,000 randomly chosen SNPs, to approximate LD under a neutral expectation. To better approximate a normal distribution, we performed a cubic root transformation on LD estimates. Using the twotPermutation function of the DAAD R package [44], we conducted permutation t-tests with 1,000 simulations to contrast average LD levels between adaptation signatures and neutral expectations. To identify long-range LD between ’peak’ SNPs with signatures of adaptation, we carried out an LD network analysis based on matrices of pairwise LD values between such SNPs. We employed three times the background LD as a threshold to identify clusters in LD networks that may reflect functional gene networks [45]. LD clusters that were located on different chromosomes but nevertheless showed increased LD among adaptation signatures were then analysed for putative functional relationships between their genes by reviewing their annotation.

### Functional annotation of adaptation signatures and gene ontology enrichment analysis

To identify candidate genes for local adaptation we queried the soybean genome annotation (Wm82.a2.v1; https://genomevolution.org/coge/GenomeInfo.pl?) and located gene models in regions with signals of adaptive differentiation or environmental associations. Using a false discovery rate of 0.05, we analyzed gene sets located in these genomic regions with ShinyGO v0.61 [46] for gene ontology enrichment, grouping the genes into functional categories defined by high-level GO terms. We also analysed gene sets belonging to different LD clusters in the LD network analysis for an enrichment of gene ontology terms. Functional effects for GmHapMap SNP markers were predicted with SnpEff [47] to categorize functional effects of the marker set. To do this, we built a SnpEff database based on the soybean reference genome and a GFF-formatted file of its genome annotation. For a functional interpretation of putative adaptation-related genes, we examined datasets that report differentially expressed genes between vegetative and generative soybean tissues (specifically comparing young leaf to flower, as well as young leaf to seed 28 days post-flowering, https://soybase.org/soyseq/heatmap/index.php). We also searched for genes responding to dehydration, salt, and drought stress [48, 49] to identify potential roles of candidate genes in developmental and adaptive processes.

### Inference of haplotype blocks

We utilized a subset of the GmHapMap SNP dataset, which includes modern European and North American cultivars as well as germplasm groups from China. This subset al.lowed us to draw comparisons between these groups, particularly at genomic regions showing genetic differentiation and environmental association signals at the haplotype level. We obtained nonoverlapping haplotype blocks using the block_calculation function from the R HaploBlocker package [50]. Subsequently, we queried the results at genomic positions that exhibit putative adaptation signals. This was done to determine the quantity, length, and composition of haplotype blocks in both genebank accessions from China and contemporary Western breeding material.

### Visualization of results

All figures were produced in R using the ggplot2 framework [51] and key extensions, such as ggrepel for label repulsion [52], gridExtra for composite layouts [53], reshape2 for data restructuring [54], ggridges for ridge density plots [55], ggpmisc for statistical annotations [56], and specialized packages like ggtree for phylogenies [57] and corrplot for correlation matrices [58].

## Results

### Population structure and reconstruction of germplasm origin

We first carried out a population structure analysis to re-establish the link between genebank accessions and their geographical origins by detecting subpopulations within the USDA soybean collection. This step was necessary for subsequent landscape genomics analyses because the majority of recorded collection locations did not correspond to the primary geographical origins of accessions (Fig. 1C). A model-based ancestry estimation of genebank accessions using SoySNP50K markers did not provide a clear criterion to determine the number of ancestral populations (*K*) in our sample of 17,017 accessions. Instead, crossvalidation using ADMIXTURE revealed that higher *K* values resulted in decreasing cross-validation error (Figure S2). Since values of *K >* 10 showed no improvement, we selected *K* = 10 for subsequent analyses. Most accessions exhibited a significant degree of genetic admixture (Fig. 1A). Confirming prior research [59, 60], we identified local subpopulations, whose composition was largely consistent with regions of origin and photoperiod sensitivity (i.e., maturity group; Fig. 1A-C). To ascertain the overall provenance of subpopulations, we focused on accessions that shared ancestry coefficients of *≥* 80% with one of the derived ancestral groups (Fig. 1D). Using this threshold, K10.2 emerged as largest group (*N* = 1,922) that mainly consisted of accessions from Japan. This group formed a distinct cluster in the PCA (Fig. 1B) and has a close genetic relationship with group K10.10 (*N* = 1,322) that predominantly consists of accessions from Korea. They mainly fall into MGs IV and V, while the accessions from Japan include MG 000 to VIII. Both groups consist of large-grained genotypes, which is a recognized characteristic of soybean traditional cultivars from Japan and Korea [61]. We further identified four groups originating from China. These groups had not been previously specified to such an extent within the USDA genebank, but their identification aligns with the known population structure of fully domesticated germplasm from China [62, 63]: Two of these groups consist of accessions primarily collected in northeastern China (K10.7; *N* = 525 and K10.8; *N* = 379). One group consists of accessions from the Huang-Huai Valley (Yellow River Valley, central China, K10.5; *N* = 269), and another group includes accessions from southern China (K10.6; *N* = 705). The latter is genetically similar to an additional group of accessions originating from India and Indonesia (K10.3; *N* = 439). The smallest group, K10.9 (*N* = 97), mainly consists of U.S. soybean varieties. This group should not be regarded as an ancestral population; rather, it represents the breeding efforts in the US during the 20th century. The origins of two other derived ancestral groups (K10.1 and K10.4) remain elusive, as no accessions with respective ancestry values of *≥* 80% were found within the germplasm sample.

For the landscape genomics analysis, we focused on the subpopulations from China because this material represents four well-separated genetic clusters (Fig. 1B). They cover the diverse agroclimatic range of soybean cultivation in this region and occupy specific subareas (Fig. 1C-E; Table 2). The subpopulations exhibit considerable intra-group variation in their MG classification, indicating the broad adaptation of germplasm within subareas.

To infer the original agro-environments, we used maturity group classifications to divide the northeastern clusters (K10.7 and K10.8) into MG000-0, MGI, and MGII-III subgroups (Table 1) and mapped them to latitudinal ranges (Fig. 1D). This method mirrors the latitudinal segmentation of soybean germplasm based on photoperiod sensitivity noted in North America and Europe [13, 64]. For K10.5 and K10.6, we avoided MG clustering because in central and southern China multiple cropping is practiced. Instead, we split the southern Chinese cluster (K10.6) by provincial origin, aligning with known ecotypes [34–36]. The Middle to Lower Reaches of Yangtze River (MLRY) group has germplasm from Anhui, Henan, Hubei, Jiangsu, and Shanghai, while the Lower Subtropical Region (LSR) includes Fujian, Hunan, Jiangxi, and Zhejiang provinces. In total, we specified eight germplasm groups spanning six growing regions (Tables 1 and 2; Figure S3). We used these groups to pinpoint genomic regions linked to agroclimatic adaptation.

### Differentiation and adaptation signatures in germplasm originating from China

We investigated the genetic architecture of soybean environmental adaptation using genetic differentiation among Chinese germplasm groups and associations of allele frequencies with environmental gradients. Using GmHapMap SNPs, we considered two scenarios: Scenario A encompassed six germplasm groups from northeastern to southern China, while scenario B included five groups from the same northsouth transect. They differed with respect to the northeastern groups and included K10.7 for scenario A and K10.8 for scenario B, respectively (Fig. 1B-C; Table 1). To study adaptation in both scenarios, we employed the first two principal components from environmental characterization as population covariate inputs from six soybean growing regions (see Table 2). These six environments in China represent a clear latitudinal gradient from north to south, with the northern regions (Environments 1–4, 49.55°N to 36.33°N) characterized by cooler temperatures, lower accumulated heat units, higher diurnal temperature ranges, and generally lower precipitation levels. The southern regions (Environments 5–6, 321°N to 27.70°N) exhibit warmer temperatures with higher accumulated heat, lower diurnal temperature ranges, and substantially higher precipitation levels in the transition from continental to subtropical growing conditions. Due to the geographic distribution overlap of K10.7 and K10.8, but a significant genetic differentiation between the clusters included in two scenarios allowed to identify common and unique adaptation strategies between germplasm groups. These germplasm groups and their regional assignments align with prior studies of Chinese landraces highlighting distinct genetic groups tied to traditional cultivation regions [34–36, 62, 63].

Candidate genes within genomic regions showing significant adaptation signals were selected based on two key criteria: first, statistical thresholds defined by the 99% quantile of XtX values (indicating significant genetic differentiation) or Bayes Factors exceeding 10 (demonstrating strong gene-environment associations); and second, functional relevance established through soybean genome annotations, significant Gene Ontology (GO) term enrichment (FDR < 0.05), and available gene expression datasets related to developmental and stress-response processes. This approach ensures both statistical rigor and biological relevance in identifying candidate genes underlying environmental adaptation.

**Fig. 2:**
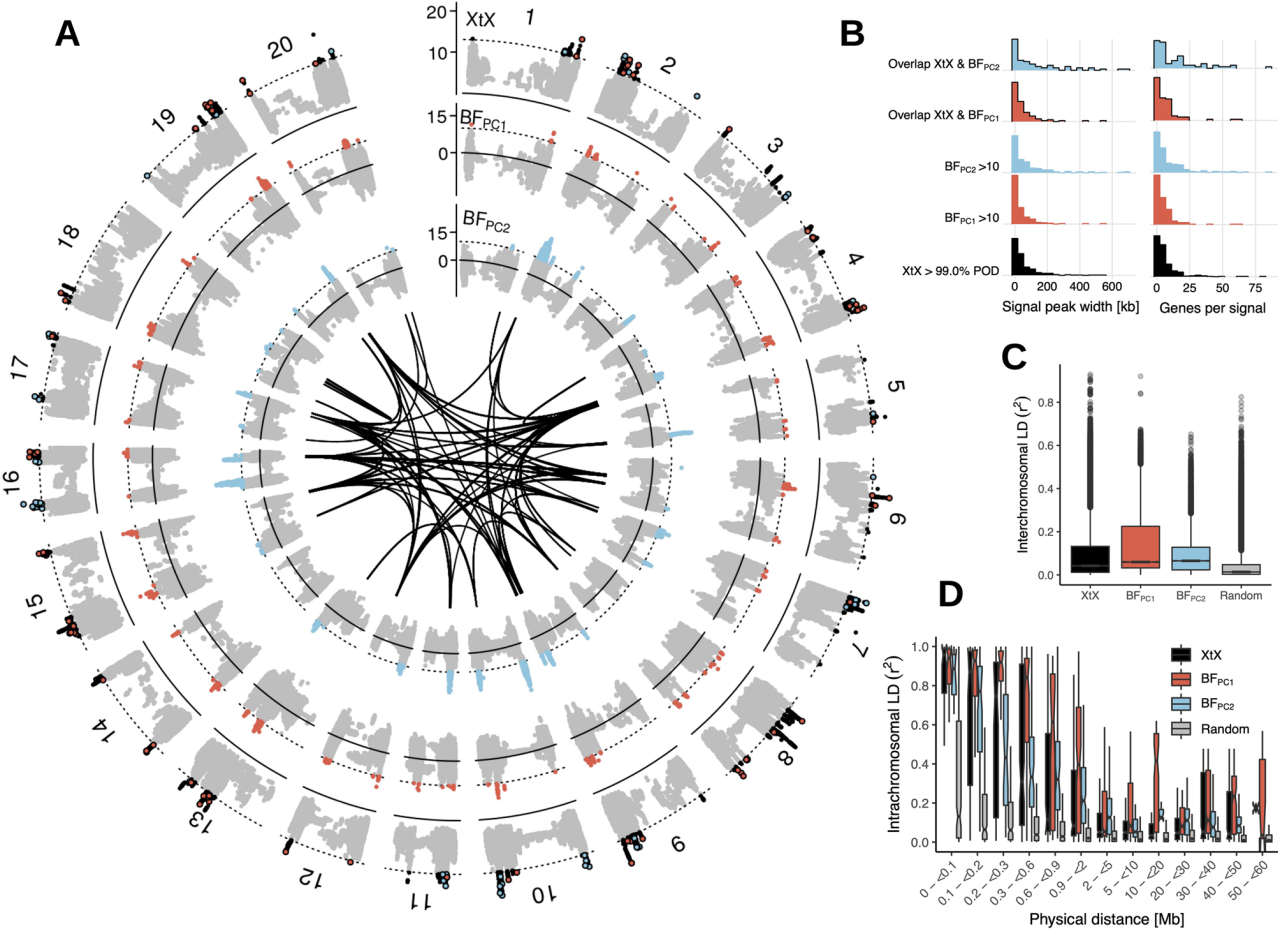
Result summary of the genome-wide scan for adaptation signatures. (**A**) Manhattan plot of the *XtX *statistic and of genotype-environment associations with the first two environmental principal components in Bayes factors for all 20 chromosomes in scenario A (for scenario B see Figure S5). Red and blue points in the *XtX *track represent overlaps between genetic differentiation and association signals with the first and second environmental principal components. The dotted horizontal lines represent the 1% POD significance threshold of the *XtX *statistic and the threshold of BF = 10 deciban. Black lines in the center indicate regions with elevated LD between adaptation signatures exceeding the level of background LD three-fold and a minimum physical distance of 5Mb. (**B**) Width of genomic regions with adaptation signatures and number of candidate genes. (**C**) LD between regions with adaptation signatures from different chromosomes in genetic differentiation and association signals and between randomly selected SNPs. (**D**) Decay of LD with physical distance between regions with adaptation signatures and between randomly selected SNPs

**Fig. 3:**
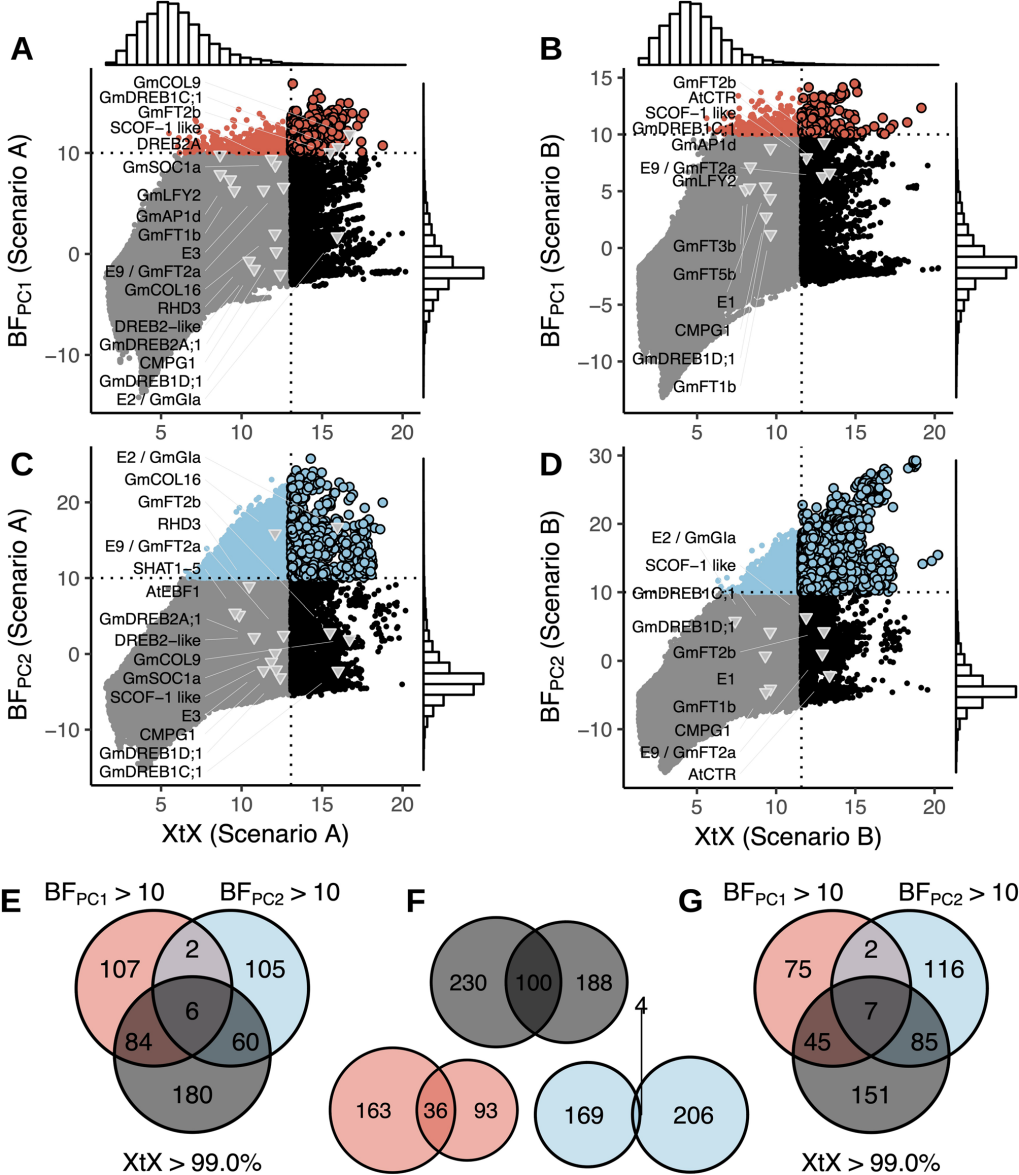
Relationships of adaptation signatures among genetic differentiation, association statistics, and scenarios: (**A-D**) Correlation between genome-wide differentiation estimates (*XtX*) and genotypeenvironment associations (BF) for the first (**A-B**) and second (**C-D**) environmental principal components in scenario **A** (**A**,**C**) and **B** (**B**,**D**). The dotted vertical lines mark the 1% POD significance threshold of the *XtX *statistic, and the horizontal lines indicate a BF threshold of 10 deciban. Known soybean genes significant for environmental adaptation, surpassing the 5% *XtX*POD significance threshold or having Bayes factors >5 decibans, are labeled to indicate putative adaptation loci meeting our significance criteria and loci in the upper bounds of the XtX and/or BF distribution. (**E-G**) Venn diagrams showing the count of genomic regions with significant differentiation or association signatures and their overlaps in scenarios **A** and **B**

Signatures of adaptive genetic differentiation appeared in both scenarios across all 20 chromosomes. Here, we show results for scenario A (Fig. 2) and provide the results for scenario B in Figure S5. The signatures encompass genomic regions up to 826 kb, covering 6,314 annotated genes (Figs. 2A, 3A-G and S5). 90% of these signals covered regions of *<* 150 kb length and included *<* 5 candidate genes (Fig. 2B). Scenario A identified 330 genomic regions with significant genetic differentiation among germplasm groups, while scenario B identified 288, with 100 overlapping regions between both scenarios (Fig. 3E-G). Genomic regions associated with environmental gradients (Table 2) exhibited comparable magnitudes and were identified in one or both scenarios. Between 52 and 92 significantly differentiated genomic regions (based on the *XtX* statistic) matched those from the environmental association analysis. Regions pinpointed by both methods contained genes known to be involved in soybean environmental adaptation (Fig. 3A-D).

**Fig. 4:**
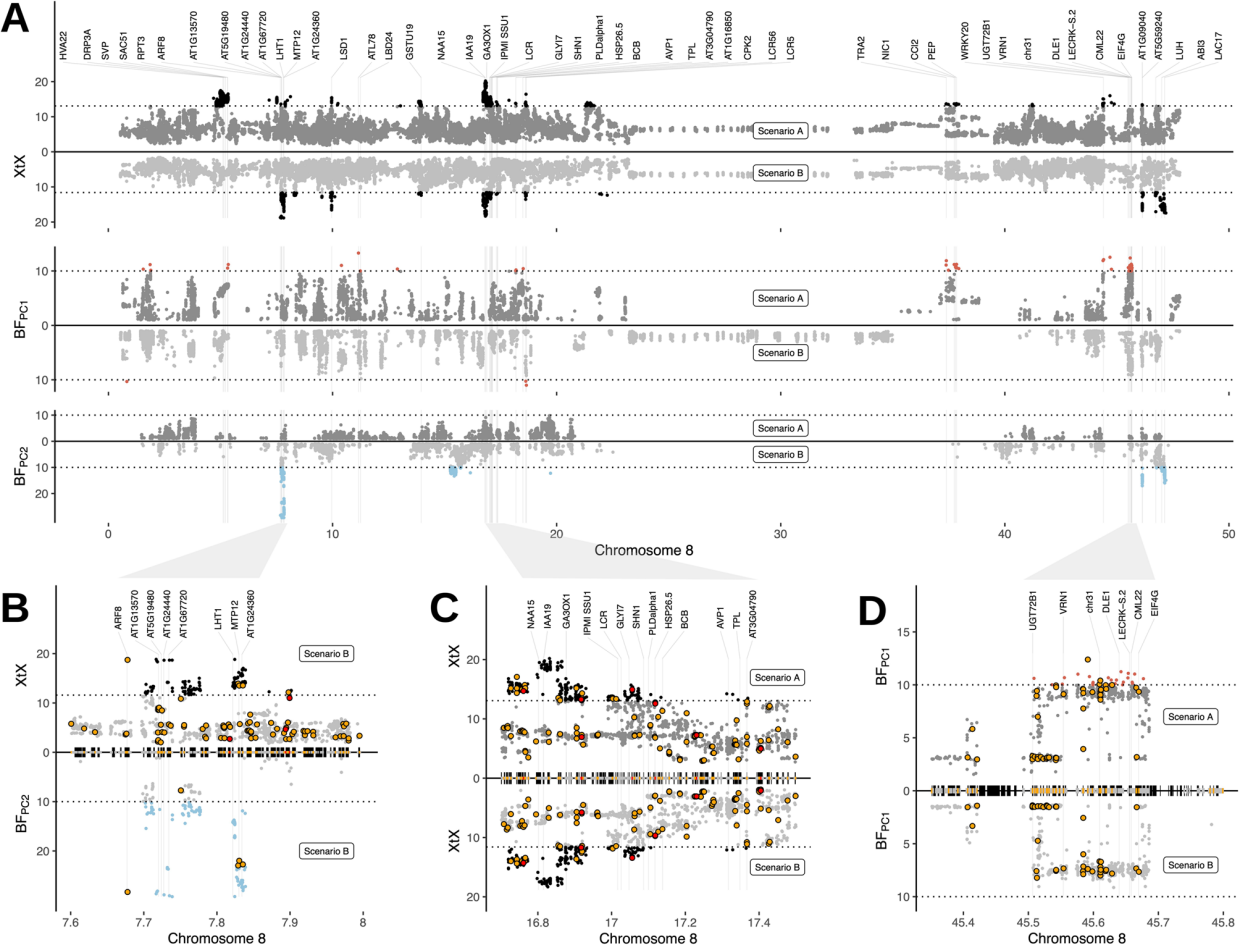
Identification of candidate genes with a putative role in environmental adaptation on chromosome 8. (**A**) Manhattan plot of the *XtX *statistic and of genotype-environment associations with the first two environmental principal components in Bayes factors for scenario **A** and scenario **B** for the complete chromosome 8. Negative Bayes factors are omitted and dotted horizontal lines are analogous to Fig. 2A. Labeled candidate genes are listed in Tab 3. The unit of the x-axis is Megabases (Mb). (**B-D**) Detailed view of genomic regions with adaptation signatures. Black blocks on the x-axis indicate the positions of predicted gene models. Red and yellow blocks and points indicate non-synonymous variants with high and moderate impacts on protein function. Further close-ups of regions with candidate genes on chromosome 8 are included in Fig. S6

A gene ontology (GO) enrichment analysis (FDR < 0.05) identified one to twenty significantly enriched GO terms in genes that are either located in highly differentiated genomic regions or are associated with environmental gradients. These GO terms are related to flowering, photoperiodism, and responses to both abiotic and biotic stresses (Table S1), consistent with a putative role in local environmental adaptation. A further examination of genome annotations further highlighted such a role. For example, chromosome 8 contains > 20 genomic regions with adaptation signatures that harbor 64 genes with homologs in other species (Figs. 4 and S6). Based on their sequence homology, many of these genes likely play roles in environmental adaptation, such as regulating flowering, responding to abiotic stress, and nutrient uptake (Fig. 4A; Table 3).

**Table 3:** Overview of candidate genes located in genomic regions with adaptation signatures on chromosome 8 (as detailed in Fig. 4) and evidence for their putative involvement in environmental adaptation. The relationship between gene designation, gene model and evidence for gene function was obtained from SoyBase. Information on the physical position of the gene models is included in Worksheet 6 in the Supplementary Data. Gene designation corresponds to gene labels in Fig. 4. *At*: *Arabidopsis thaliana*

Gene designation	Gene model	Evidence for gene function in local adaptation
*HVA22*	*Glyma.08g066700*	Role in vesicular traffic induced by environmental stresses such as dehydration, salinity and extreme temperatures in barley
*DRP3A*	*Glyma.08g067100*	Involved in mitochondria fission and crucial for cold induced mitochondria fission in *At*
*SVP*	*Glyma.08g068200*	Floral repressor functioning within the thermosensory pathway in *At*, represses *FT *expression
*SAC51*	*Glyma.08g069700*	Involved in xylem development through stress induced thermospermine regulation in *At*
*RPT3*	*Glyma.08g069800*	Involved in phototropism in *At*
*ARF8*	*Glyma.08g100100*	Auxin response factor involved in lateral root development in *G. Max*, involved in stress-related auxin signalling and regulation of flower maturation in *At*
*AT1G13570*	*Glyma.08g100500*	Differentially expressed in response to abiotic stresses in *At*
*AT5G19480*	*Glyma.08g100600*	Differentially expressed in response to abiotic stresses and between vegetative and reproductive stages in *At *and rice
*AT1G24440*	*Glyma.08g100700*	Differentially expressed in response to abiotic stresses in *At*
*AT1G67720*	*Glyma.08g100800*	Involved in root nodule symbiosis in *Lotus japonicus*
*LHT1*	*Glyma.08g101900*	Amino acid transporter in *At*, possibly involved in nitrogen acquisition
*MTP12*	*Glyma.08g102000*	Zinc transporter in *At*
*AT1G24360*	*Glyma.08g102100*	Involved in fatty acid synthesis and lipid metabolism, may function in stress response signaling
*LSD1*	*Glyma.08g129400*	Involved in responses to water stress, vegetative biomass production and generative development
*ATL78*	*Glyma.08g146600*	Regulation of cold and drought stress responses in *At*
*LBD24*	*Glyma.08g147300*	Members of the *LBD *gene family exhibit differential expression in response to various stresses in soybean
*GSTU19*	*Glyma.08g174600* to Glyma.08g174900	Involved in tolerance to salt and drought in *At*
*NAA15*	*Glyma.08g207800*	Abscisic acid mediated drought stress responses in *At*
*IAA19*	*Glyma.08g207900*	Auxin-sensitive maintainer of glucosinolate levels that affect drought tolerance in *At*, possibly via stomata regulation
*GA3OX1*	*Glyma.08g208300*	Synthesis of bioactive gibberellins that have regulatory roles in development, cold sensitivity of male meiosis in *At *and rice
*IPMI SSU1*	*Glyma.08g210500*	Jasmonate responsive mediation of environmental stresses in *At*
*LCR*	*Glyma.08g210700*	Response regulation to abscisic acid, salinity and drought in *At*
*GLYI7*	*Glyma.08g211100*	Upregulated in response to salt, osmotic stress and wounding in *At*
*SHN1*	*Glyma.08g211600*	Involved in wax cuticle biosyntesis, affects tolerance to heat and drought in *At*
*PLDalpha1*	*Glyma.08g211700*	Involved in abscisic acid and salt stress response in *At*
*HSP26.5*	*Glyma.08g212000*	High temperature-inducible heat shock protein in *At*
*BCB*	*Glyma.08g212500*	Cold responsive gene involved in cold acclimation in *At*
*AVP1*	*Glyma.08g214300*	Involved in drought and salt stress tolerance in *At*
*TPL*	*Glyma.08g214600*	Involved in stress responses and regulatory cascades that control flowering time in *At*
*AT3G04790*	*Glyma.08g214700*	Downregulated in response to drought in an *At *relative
*AT1G16850*	*Glyma.08g224100*	Involved in response to cold and other environmental stresses
*CPK2 / AT3G10660*	*Glyma.08g227200*	Calcium-dependent protein kinases are involved in abiotic stress tolerance regulation
*LCR56 / AT3G20993*	*Glyma.08g228400*	Low-molecular-weight-cysteine-rich, adaptive function unclear
*LCR5 / AT2G28355*	*Glyma.08g228600*	Low-molecular-weight-cysteine-rich, adaptive function unclear
*TRA2 / AT5G13420*	*Glyma.08g277700*	Involved in lignification in *At*
*NIC1 / AT2G22570*	*Glyma.08g279200*	Involvement in ABA-mediated stress responses is hypothesised
*CCl2 / AT4G38060*	*Glyma.08g279400*	Increased expression in response to salt, osmotic, cold and drought stress
*PEP / AT5G10480*	*Glyma.08g279700*	Involved in flowering via RNA processing of *FLC *in *At*
*WRKY20*	*Glyma.08g325800*	Expression of *G. soja WRKY20 *in *At *enhances drought tolerance and regulates ABA signalling
*UGT72B1 / AT4G01070*	*Glyma.08g338100* to Glyma.08g338900	Stress-responsive glycosyltransferase in *At *and other *Brassica *species
*VRN1*	*Glyma.08g339100,*	Soybean homologs of *AtVRN1 *are responsive to cold and participate in the photope- *Glyma.08g339200 *riodic regulation of flowering
*Glyma.08g339500*	*Glyma.08g339500*Coexpressed with genes in flower specific coexpression subnetwork (Phytozome 12)	Coexpressed with genes in flower specific coexpression subnetwork (Phytozome 12)
*chr31 / AT1G05490*	*Glyma.08g339800, * *Glyma.08g339900*	Regulation of stress-related signalling
*DLE1 / AT3G60190*	*Glyma.08g340200*	Involved in cold acclimation in *At*
*LECRK-S.2 / AT2G32800*	*Glyma.08g340300*	Differentially expressed in response to cold, salt and osmotic stress
*CML22 / AT3G24110*	*Glyma.08g340400*	Regulation of stress-related signalling
*EIF4G / AT3G60240*	*Glyma.08g340500*	Involved in flowering in *At*
*AT1G09040*	*Glyma.08g346400*	Upregulated under nutrient limiting conditions in *At*
*AT5G59240*	*Glyma.08g346500*	Upregulated in response to salt stress in *At*
*LUH / AT2G32700*	*Glyma.08g354500*	Regulator of abiotic stress response genes in *At*
*ABI3 / AT3G24650*	*Glyma.08g357600*	Involved in flowering regulation and in response to abiotic stress in *At*
*LAC17 / AT5G60020*	*Glyma.08g359400*	Involved in lignification in *At*

**Fig. 5:**
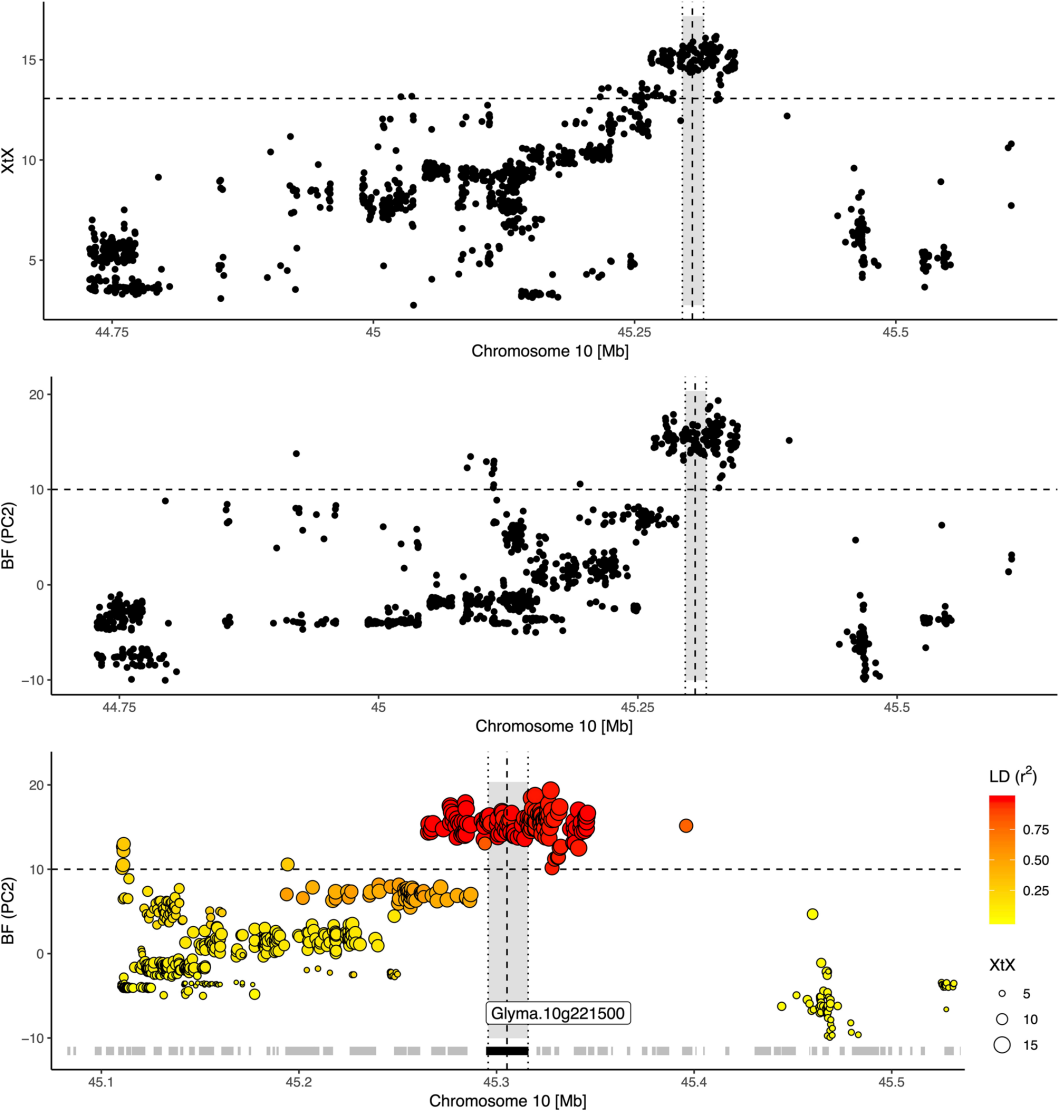
Differentiation and association signature on chromosome 10 highlighting the region that harbors the*E2 *locus (indicated by vertical dotted line) observed in scenario **A**. Bottom: Close-up of the region. Grey blocks on the x-axis indicate the position of predicted gene models, the black block indicates the *E2 *locus. Pairwise LD is reported with respect to a central marker in the *E2 *locus

We used SnpEff to predict the potential functional effects of non-synonymous variants. Only 0.3% of SNPs were classified as having a strong functional impact (e.g., loss of function), while 5.7% were considered to have moderate effects, such as changes in protein effectiveness [47]. This data can help prioritize candidate genes with non-synonymous variants in genomic regions with adaptation signatures, potentially including causative variants (Figs. 4B and S7). However, identifying causative genes, especially closely linked ones (Fig. 4C-D), requires further validation. Many regions showing adaptation signatures lacked non-synonymous variants among the statistically supported SNPs for selection (Figure S7), implying that our SNP set may be linked to, but not include, causal variants. Since a substantial proportion of genomic regions emerged in more than one selection test, they may constitute robust signals [5]. For instance, on chromosome 8, we identified genomic regions with significant differentiation in both scenarios A and B (e.g. Fig. 4C), as well as regions with strong genetic differentiation and association to environmental variables (e.g. Figs. 4B and S7). Adaptation signatures on other chromosomes were consistent with those on chromosome 8 (Figs. 2 and S6). The Supplementary Data include all 6,314 genes in regions with putative adaptation signatures. When compared with earlier studies, several previously known adaptation genes did not meet our significance thresholds (Fig. 3A-D), suggesting that our method is conservative. The maturity loci *E3* and *E1* for example showed differentiation between germplasm groups but lacked statistical significance, while maturity loci *E2* and *E9* displayed robust adaptation signatures (Figs. 3A-D and 5). The low level of differentiation at the *E1* locus in Chinese germplasm is likely attributable to the low frequency of the rare *e1* allele, even in regions like northeastern China where it is presumed beneficial [65, 66]. Such findings emphasize that outcomes from landscape genomic studies are inherently population-centric. Additionally, genome scans might occasionally overlook polygenic or functionally redundant traits [5].

### Linkage disequilibrium among genomic regions with adaptation signatures

Many genomic regions with differentiation or association signatures exhibited increased mean pairwise LD (*r*^2^) between SNPs. Pairwise *r*^2^ values for peak markers in different regions with signatures of adaptation consistently exceeded random genome-wide marker pairs (Two-sample permutation t-test; *p <* 0.001). This was observed both within and between chromosomes (Fig. 2C-D). Background LD between markers on different chromosomes averaged *r*^2^ = 0.21. This LD level was exceeded two to five times more often by markers with adaptation signatures on separate chromosomes. For higher *r*^2^ values, this ratio increased by five-fold in scenario B and 38-fold in scenario A (Table S2). High LD levels between unlinked regions (Figs. 2A and S5) suggest epistatic selection of polygenic traits for local environmental adaptation [45].

**Fig. 6:**
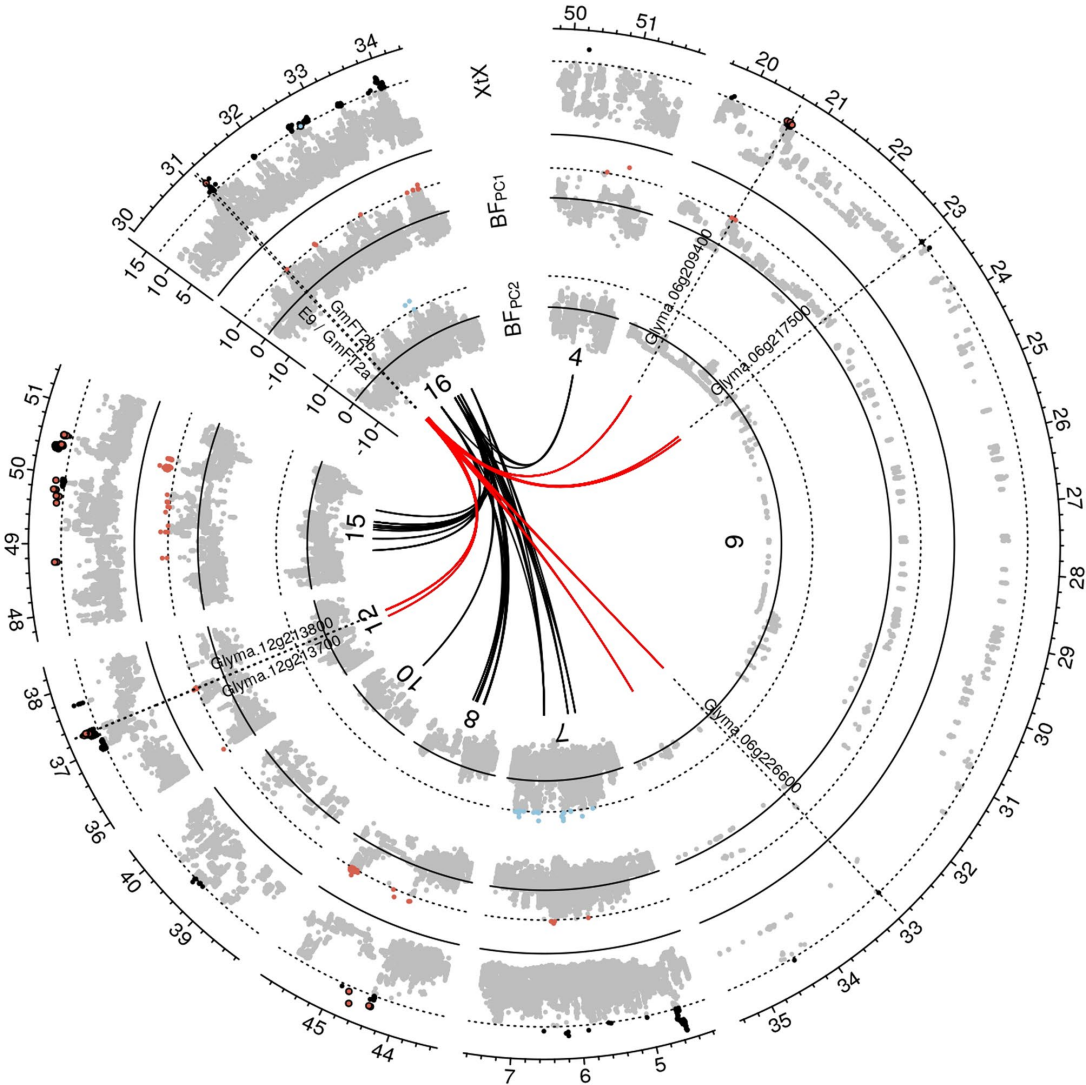
Linkage disequilibrium networks among genomic regions with adaptation signatures. Detailed view of a Manhattan plot corresponding to Fig. 2A for genomic regions that display LD>3-fold background LD with a region on chromosome 16 that includes the genes*E9 */ *GmFT2a *and *GmFT2b *which are involved in the regulation of flowering. Only outgoing LD connections (from chromosome 16) are displayed in form of black and red lines. Red lines indicate putative members of a network involved in flowering regulation

**Fig. 7:**
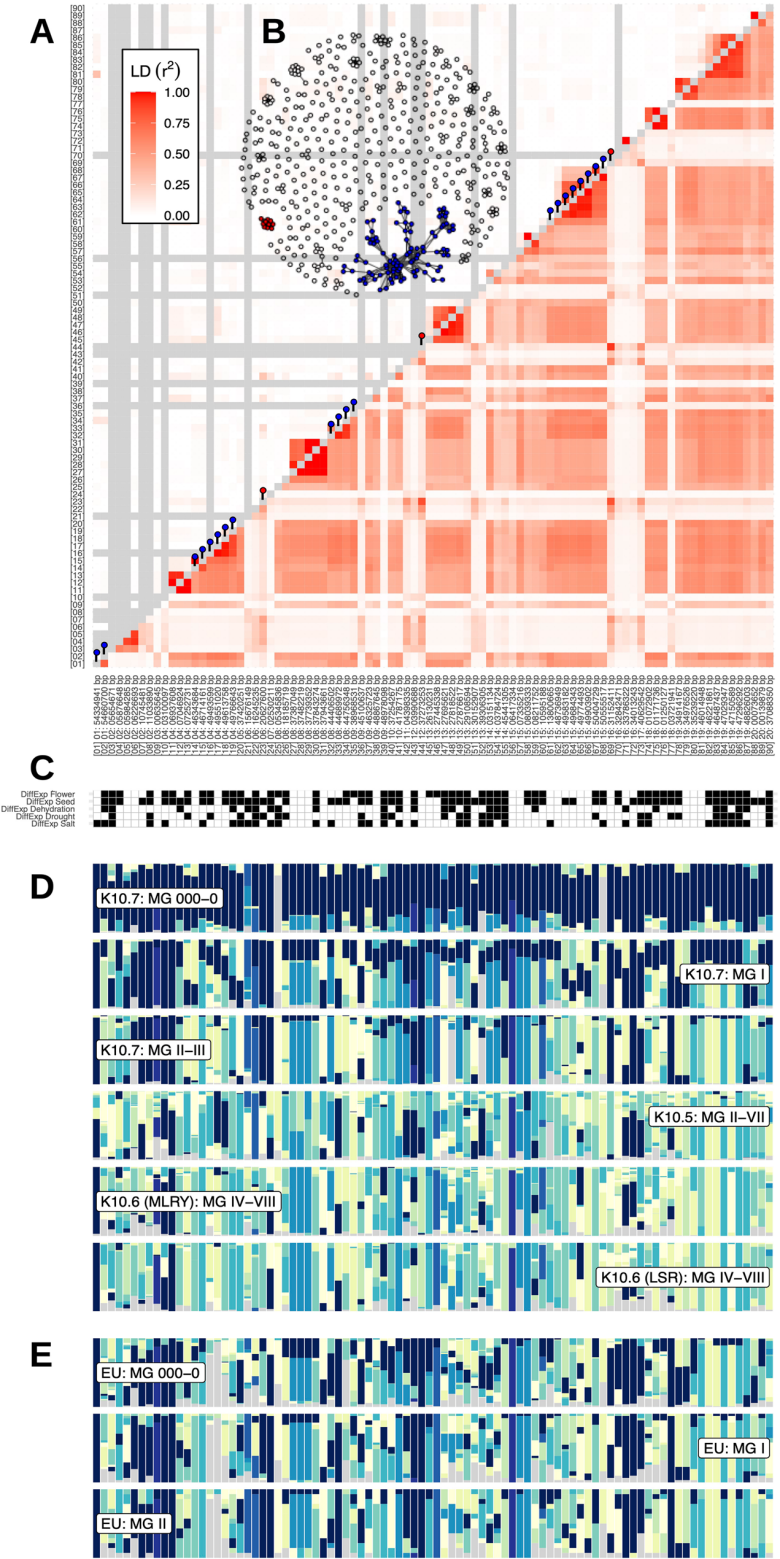
Comparison of haplotype block proportions and of LD between genomic regions with adaptation signatures in germplasm from China and in modern European varieties. (**A**) LD estimates among 90 genomic regions with overlapping genetic differentiation and genotype-environment association signatures (PC1) observed in scenario **A** for germplasm groups from China (below diagonal) and in modern European soybean varieties (above diagonal). Red and blue pins on the diagnonal indicate regions that clustered in LD network analysis. For a complete overview of LD among all selection signatures in scenarios **A** and **B** see Figures S7 and S8. (**B**) LD network of 544 genomic regions with selection signatures in scenario A for germplasm groups included in scenario A. Two clusters (92 and 10 regions, respectively) comprising LD connections exceeding the level of background LD 3-fold and a minimum physical distance of 5Mb are highlighted in blue and red. (**C**) Differentially expressed genes during development and in response to abiotic stresses are indicated in black for the displayed 90 genomic regions. (**D**) Haplotype block proportions in germplasm groups from China in the 90 genomic regions mirror a latitudinal gradient. (**E**) Haplotype block proportions in modern European varieties in the 90 genomic regions reveal an underrepresentation of haplotype blocks of North-Eastern Chinese descent that could harbour adaptive variation to high-latitude cold regions

In our LD network analysis of genomic regions with adaptation signatures, 3-fold background LD was used as threshold to define putative functional clusters. Both scenarios A and B revealed LD clusters, primarily within chromosomes (Figs. 7A-B, S7 and S8). Given that LD clusters can emerge from strong selective sweeps, inversions inhibiting recombination [43], or neutral variation in recombination rates [67], we focused on LD clusters with a minimum distance of 5 Mb and with interchromosomal LD connections because they may represent functional epistatic interactions. In scenario A, two such instances of interchromosomal LD clusters with adaptation signatures (Figs. 7 A-B and S8) and in scenario B, three such clusters were observed (Figure S9). These LD clusters were not enriched for GO terms. One cluster in scenario A included a soybean homolog of *AtFT2b* [68] and the flowering and maturity locus *E9*, which is a soybean homolog of *AtFT2a* [69]. Both are located on chromosome 16 in close proximity and this region was in high LD with regions on chromosomes 6 and 12 (Fig. 6). These regions also show adaptation signatures and contain candidate genes with putative roles in flowering regulation. For example, gene *Glyma.06g209400* has the GO term *regulation of flower development*, and the *Arabidopsis* homolog of *Glyma.12 g213700* (*AT4G21200*) is involved in initiation of flowering [70]. Other candidates of this cluster may regulate heat stress response during flowering: The homolog of *Glyma.06g226600* (*AT3G14200*) is a stress-responsive gene involved in pollen tube growth in *Arabidopsis* [71], while *Glyma.12 g213800*.

(*AT3G01140*) is a regulator of cuticle biosynthesis that is inducible in petals by heat stress [72].

To further investigate functional connections between genes in LD clusters, we examined datasets of genes differentially expressed between vegetative and generative tissues or in reaction to abiotic stresses [48, 49]. Numerous differentially expressed genes are located in putative adaptive genomic regions (Fig. 7C). However, we observed that LD clusters identified in genebank accessions from China are not present in US and European cultivars (Figs. 7A, S8 and S9), suggesting that adaptive variation or epistatic interactions in the former may not be present in the latter group. In summary, LD network analysis identifies gene clusters among genomic regions with adaptation signatures located on different chromosomes. Robust evidence for functional interactions of genes in distant genomic regions with strong LD remains scarce except for the genetic network involving maturity locus *E9* and genes regulating flowering time.

### Frequency of putative adaptive haplotypes in U.S. And European cultivars

To utilize genomic regions with adaptation signals for breeding elite varieties, we identified shared haplotype blocks between germplasm from China and modern breeding material from North America and Europe. In Canada and Central Europe, soybean breeding aims for adaptation to cooler regions at higher latitudes, for which little genetic variation is currently available [73–75]. New variation for cold adaptation may be found in landraces that are adapted to the far north of northeastern China and adjacent regions in Russia [20, 76]. Since haplotype blocks in adaptive genomic regions of germplasm from China exhibited a latitudinal gradient (Figs. 7D and S9-S12), we investigated whether they are present North American and European varieties. We found variable proportions of haplotype blocks with ancestry from northeast China in these varieties (Figs. 7E and S9-S12). For example, the maturity locus *E9* in the earliest maturing modern North American and European cultivars (MG 000–0) was nearly fixed for the haplotype block prevalent in material from northeastern China, whereas haplotype blocks with ancestry in material from Southern China are more frequent in cultivars with later maturity. We observed similar patterns in other genomic regions, which likely results from selection for improved adapation to specific latitudes. This interpretation is also consistent with the higher frequency of haplotype blocks originating from northeastern China in modern versus early-maturing cultivars (Figures S9-S12). Overall, adaptive haplotypes of northeastern ancestry were more common in early-maturing than in later-maturing cultivars, but they are still underrepresented in many genomic regions of North American and European varieties. Therefore, there is a strong potential for further introgression of potentially adaptive alleles for cultivation in cool high latitude regions. Haplotype profiles of modern early-maturing North American and European cultivars (Figures S13 and S14) indicate that integration can be achieved within breeding material through targeted crosses and marker-assisted selection, focusing on blocks from northeastern China that are associated with local adaptation.

## Discussion

We conducted landscape genomics analyses on soybean genebank accessions originating from China, which is part of the USDA Soybean Germplasm Collection [21]. Similar to studies in other crops [77–80], we identified numerous candidate loci with adaptation signatures (Figs. 2A and 3), including genes involved in flowering control, abiotic stress response, and nutrient acquisition. The latter is likely an adaptation to varying soil nutrient levels across different climates [81, 82]. Flowering time is a key diversification trait because it influences adaptation based on photoperiodic sensitivity [62]. In total, our differentiation scans revealed 6,314 genes that may contribute to local adaptation. Several of these genes displayed substantial genetic differentiation and significant associations with environmental variables (Fig. 3E-G, Supplementary Data Worksheet 6). They include candidate genes for abiotic stress response based on their role in stress response pathways that exhibit divergent expression under drought, salt, dehydration, or cold stress conditions (Fig. 7C; Table 3). These genes can be prioritized for further functional investigations or marker-assisted selection in breeding programs. For instance, breeding programs in Canada and Central Europe targeting adaptation to high latitude cold regions could greatly benefit by introgressing putative adaptive regions identified in scenario A, utilizing germplasm from the northernmost areas of soybean cultivation in China and Russia (Table 2).

However, genome scans are subject to confounding factors that can either produce false positives or obscure genuine signatures of adaptation, leading to false negatives. Such factors encompass population structure, demographic history, data limitations, and statistical constraints [5]. In the following, we discuss the influence of these factors on our findings, highlighting current constraints and possible strategies for the functional validation of inferred soybean adaptations.

### Geographic origin of genebank accessions

The use of landscape genomics for domesticated crops often involves analyzing germplasm from *ex situ* collections established during the Green Revolution to preserve traditional cultivars. These efforts primarily centered on collecting germplasm, neglecting the recording of critical environmental and geographic parameters to characterize the environments in which traditional cultivars evolved [83]. Such a lack of information is also evident in the USDA soybean genebank collection [21]. To acommodate this limitation, our approach differs from an earlier landscape genomics study on USDA material [18]. First, we excluded accessions from Korea and Japan because they are genetically different (Fig. 1A-D). These accessions are influenced by geographical and ethnocultural boundaries and may have been independently domesticated [61], which confound genome scans. We therefore focused on accessions from China that provided a sufficiently large sample size and environmental diversity to detect signals of local adaptation (Figs. 1E and S1). Secondly, we did not rely on the georeferences in the passport data, because they are often inaccurate [20] and were available for only a fraction of the USDA collection (only 924 of more than 6,100 accessions from China are georeferenced). To avoid bias from misallocation and uneven representation (Fig. 1C), we clustered genebank accessions by their likely regional origin based on their population structure, classification into maturity groups and provincial origin (Figure S3). Therefore, our analyses were based on allele frequency differences of genetically homogeneous groups without admixed genotypes instead of the genotype of individual genebank accessions. Excluding admixed individuals was an analysis choice to balance the comprehensive representation of germplasm and the reliability of georeferencing. This may exaggerate genomic differences between germplasm groups, especially if they are evenly distributed and adaptive mixing occurred in transition zones [18, 35, 59]. Augmenting passport data, perhaps by integrating information from the Chinese soybean germplasm collection [84], might allow for the inclusion of admixed genotypes in subsequent studies, if collections and their passport information can be combined. New collections from core cultivation areas could also provide high-quality georeferenced data for anchoring existing collections.

### Selective forces shaping soybean germplasm

We utilized EARTHSTAT [31] and WorldClim [32] datasets, assuming they adequately approximate the geographical and environmental conditions during historical soybean adaptation. For the association analyses we considered a limited number of environmental parameters (Table 2), which were derived from three environmental attributes: temperature, precipitation, and solar radiation. Although such an environmental characterization is not exhaustive, prior research on soybean ecotypes [34, 36] suggests that these parameters include the main selective gradients. By focusing on large-scale comparisons of origin within germplasm groups (Fig. 1E), we ensured the spatial accuracy of our data. The temporal resolution is limited to monthly averages and does not account for abrupt weather changes because they are not captured by monthly summaries [5, 85]. The pronounced multicollinearity of the climate data (Figure S4) complicates the identification of causal environmental parameters for environmental adaptation. To account for correlations among environmental parameters, we condensed them via principal components analysis and subsequently used the first two principal components, which captured 97.6% of the variance for genotypeenvironment correlations (Table 2). Even with these constraints, we identified significant associations in nearly half of the genomic regions that additionaly showed strong genetic differentiation (high *XtX* values) between germplasm groups (Fig. 3E-G). This result suggests that our approach is very promising for future studies aiming to identify causal genes or genomic regions for local adaptation.

Besides natural selection, native soybean germplasm was subjected to multi-faceted human-driven selection after domestication. This selection targeted aesthetic and culinary traits like colour, texture, and flavour, in conjunction with agronomic traits that were selected by local farming practices and traditional uses. Although these factors likely impacted the distribution and genomic diversity of traditional cultivars, they are not documented in passport data. Disentangling the genetic changes resulting from environmental adaptation from those originating from from agricultural selection is challenging and suggest a careful interpretation of our results.

### Accounting for population structure and demography

The pronounced average genetic differentiation among germplasm groups amplifies the variance in differentiation metrics such as *XtX*. Consequently, only loci with substantial selection effects are classified as significant outliers, either based on *XtX* distributions or through elevated Bayes Factors in environmental association studies [5, 86]. As a result, our method leans towards recognizing loci with prominent effects on adaptation. This bias likely accounts for the omission of some established adaptation genes in our selection tests (Fig. 3A-D). Furthermore, neutral population processes, including migration, dispersal, and bottlenecks, can mimic signatures of selection, potentially generating false positives in genome scans. To reliably distinguish between selection and neutrality, we employed BAYPASS utility functions. By estimating allele frequency covariance among populations, we established a null distribution of *XtX*, thereby reducing false positives [37]. Another method would have involved modeling the demographic history of soybean in China to simulate the *XtX* statistic. However, this technique is prone to errors originating from inaccuracies in its demographic history [5], particularly considering the complex evolution of this crop influenced by human selection, introgression from wild *G. soja*, and multiple domestication events [61]. Access to highquality genome sequences from traditional cultivars will enable a more precise analysis of the demographic history of this crop.

### Genetic architecture of environmental adaptation

Local adaptation may result from minor and correlated changes in allele frequencies across multiple loci with small effect [87]. Under such a polygenic mode of adaptation, most loci involved are expected to lack signatures of strong selection and are not identified in genome scans. Such a scenario is plausible for soybean because different flowering time genes were implicated in adaptation to low versus high latitudes [11] As noted above, our observation that known genes for soybean adaptation were strongly differentiated or showed environmental association but did not exceed significance thresholds (Fig. 3A-D) is consistent with a model of polygenic adaptation. Additional factors, such as allelic heterogeneity, conditional neutrality, pleiotropy, and epistasis, can further mask adaptation signals in genome scans [5]. The prevalent self-fertilization in soybean might also limit the detection of adaptive variation, as this mode of reproduction often results in adaptations with smaller effect sizes spread across the genome [86]. Nonetheless, our study highlighted several genomic regions containing genes previously associated with soybean adaptation (Fig. 3A-D) or known for functions like flowering, photoperiodism, and stress responses (Table S1).

In a genome-wide association study of flowering times in cultivars from China and the USA that comprised different maturity groups, Wu et al. [88] identified 18 candidate genes with a putative function in flowering time, four of which (*PFT1*,* FT2a*,* FT2b*,* VRN1*) were also included in our list of genes located in adaptive genomic regions (Supplementary data 1). Other GWAS studies have identified genes with a putative role in regional adaptation, such as *FLOWERING LOCUS T 5b* (*FT5b*) or the flowering time regulator *qFT13-3*, which is likely involved in soybean adaptation to high latitudes [89] but were missed by our analysis. Viana et al. [90] used measures of genetic differentiation (*F*_*ST*_), allele frequency spectrum (Tajima’s *D*), and extended haplotype heterozygosity (EHH) to identify genomic signatures of selection in ancestral, intermediate, and elite U.S. soybean germplasm. They identified 292 genes with selection signatures, of which 61 genes (20%) were also present in our set of 6,314 genes located in genomic regions with an a signature of adaptation, which is almost twice as many as expected from a random sampling of the approximately 58,000 genes in the soybean genome (ˆ*µ*_*simulated*_ = 0.32, *p <* 0.0001). These comparisons of studies indicate that environmental association, phenotypic GWAS, and selection scans identify major adaptation genes such as *VRN1*, but also many novel candidate genes that likely reflect polygenic adaptation.

Certain genomic regions may appear adaptive in environmental association tests if they combine multiple loci of minor effects (Fig. 4B-D), although such genetic differentiation could be neutral, resulting from inversions that suppress recombination. Such, inversions can be integral to adaptation by conserving beneficial allele combinations [91]. Various adaptive regions across different chromosomes are implicated in LD networks, indicating epistatic interactions influencing fitness (Figs. 7B, S7, and S8). We identified one network that may be involved in regulating flowering (Fig. 6), determining the roles of others is challenging due to gene density, which complicates the distinction between actual adaptive targets and linked genes. The lack of enriched GO terms implies these networks could emerge from population dynamics or structure, such as allele introgressions from wild *G.soja* into East Asian landraces [61], potentially facilitating local adaptation during soybean domestication and expansion. Epistatic interactions could also be reflected in gene coexpression clusters [92]. Future studies should employ the increasing transcriptome data from native Chinese germplasm [81] to analyze correlations between gene expression variation and. LD patterns, while considering the context-specific expression of adaptation genes [93].

### Future perspectives for functional analysis and breeding

We based our analyses on SNP marker genetic variation, augmented by the GmHapMap [26], facilitating analysis to a gene-level resolution and surpassing the constraints of the SoySNP50K array [23]. Structural variants, like copy number variants or inversions, also play roles in adaptation [94]. Thus, observed adaptation patterns in larger genomic regions with high local LD (Figs. 2, S7, and S8) could indicate inversions, requiring validation via long-read sequencing. The soybean pangenome revealed numerous stressresponse genes in its dispensable segments [81], emphasizing the significance of presence-absence variation in local adaptation. Recent genotyping and sequencing projects of Russian, East Asian and European elite material [61, 95, 96] in combination with new reference genomes [81, 97, 98] anticipate a more complete pangenome that will enhance landscape genomics analyses and allow to infer the role of complex genetic variants.

Despite some constraints in our design, we identified known and new candidate genes for local soybean adaptation. Using comparative genomics, further investigation of these genes can be prioritized based on evolutionary conservation and anticipated phenotypic effects. For empirical validation, genome editing provides methods to induce mutations or adjust gene expression [99]. For instance, knock-outs of soybean homologs of the *Arabidopsis thaliana* gene, *AtLNK2*, accelerated flowering under prolonged daylight [100]. Genome-editing of the maturity genes *E1* and *E1Lb* showed significant effects on maturity and enabled the cultivation of subtropical cultivars in temperate regions. Bai et al. [101] demonstrated multiplex CRISPR/Cas9 editing, targeting 102 genes and achieving multiplex editing of eight genes in a single plant. Such plants with mutated adaptation genes can enrich both functional elucidation and soybean breeding.

Given the polygenic nature of adaptive traits, marker-assisted selection of offspring from crosses between modern breeding materials with complementary adaptive haplotypes, such as identified in our analysis of recent varieties, can provide valuable information for breeding programs aimed at adapting soybean to cooler and higher-altitude regions in North America and Europe [102]. A genomics-driven allele-stacking approach focused on elite varieties also helps to avoid the linkage drag often observed when introgressing exotic genetic resources. The large number of genomic regions associated with environmental adaptation suggests that genomic prediction could be a powerful tool for predicting polygenic adaptive traits in breeding programs. Integrating landscape genomic techniques through genomic prediction of adaptive traits is therefore a promising strategy for adapting crops to future climates and realizing the environmental benefits of soybean cultivation, for example in Europe [103]. However, such approaches require further validation, as the inclusion of genetic variants identified by genome–environment association (GEA) analyses or the use of environmental data from the site of origin does not necessarily improve the prediction of fitness-related quantitative phenotypes in traditional maize cultivars [104].

## Supplementary Information

Supplementary Material 1.

## Data Availability

The datasets generated and/or analysed during the current study are available in the following repositories. SoySNP50K genotype data for 160 modern European soybean varieties are available at http://doi.org/10.5281/zenodo.6126368. SoySNP50K genotypes of the USDA Soybean Germplasm Collection [22, 23] are available on SoyBase (https://soybase.org/snps/). GmHapMap genotypes of the USDA Soybean Germplasm Collection [26] are available on figshare (https://figshare.com/articles/dataset/USDA_20K_samples_Imputed_from_GmHapMap/7388750). Supplementary Data that include ancestry proportions inferred with ADMIXTURE and selected passport information, allele counts for soybean populations from China as inputs for BAYPASS, mean and standard deviation of marker specific XtX estimates, Bayes factors of environmental associations and a list of all candidate genes within genomic regions with putative adaptation signatures are available at http://doi.org/10.5281/zenodo.6126368.

## Abbreviations

GO: Gene Ontology
LG: Linkage disequilibrium
MG: Maturity Group
SNP: Single Nucleotide Polymorphism
