# Supplementary material for: Using landscape genomics to infer genomic regions involved in environmental adaptation of soybean genebank accessions
Source: BMC Plant Biol. 2025 Sep 1;25:1175. doi: 10.1186/s12870-025-07202-5 (PMC12400605; doi:10.1186/s12870-025-07202-5)
Supplement: Supplementary file 1 — Supplementary Material 1. [file 12870_2025_7202_MOESM1_ESM.pdf]

# – Supplementary Information –

Using landscape genomics to infer genomic regions involved in environmental adaptation of soybean genebank accessions

Max Haupt and Karl Schmid
University of Hohenheim, Stuttgart, Germany

Contents

1 Supplementary Tables **2**

2 Supplementary Figures **5**

1 Supplementary Tables

Table S 1: Results of the Gene Ontology Enrichment Analysis conducted with ShinyGO v0.61 (http://bioinformatics.sdstate.edu/go/) for genes located in selection signatures.

Signal Type and Scenario	Enrichment FDR	Genes in list	Total genes	Functional Category
XtX A	3.00E-03	18	127	Response to reactive oxygen species
	2.60E-02	71	1125	Cellular component assembly
	2.60E-02	11	69	Response to hydrogen peroxide
	2.60E-02	18	164	Response to osmotic stress
	2.60E-02	10	60	Protein complex oligomerization
	3.20E-02	17	157	Response to salt stress
	3.20E-02	6	23	Phloem development
	3.20E-02	4	9	Long-day photoperiodism
	3.20E-02	4	9	Long-day photoperiodism, flowering
	3.20E-02	4	9	Regulation of long-day photoperiodism, flowering
	4.00E-02	56	888	Protein-containing complex assembly
BF_{PC1} A	2.30E-02	6	40	Jasmonic acid metabolic process
	2.30E-02	6	35	Salicylic acid metabolic process
	2.30E-02	5	23	Phloem development
	2.30E-02	6	41	Phenol-containing compound metabolic process
	2.90E-02	7	63	Benzene-containing compound metabolic process
	3.00E-02	6	47	Phloem or xylem histogenesis
	3.00E-02	61	2059	Cellular catabolic process
	3.00E-02	63	2140	Organic substance catabolic process
	3.00E-02	7	67	Aspartate family amino acid biosynthetic process
	3.00E-02	2	2	Gamma-aminobutyric acid catabolic process
	3.20E-02	6	53	Stomatal movement
	3.20E-02	6	53	Regulation of stomatal movement
	3.40E-02	5	36	Methionine biosynthetic process
BF_{PC2} A	2.70E-04	9	36	Ionotropic glutamate receptor signaling pathway
	2.70E-04	9	36	Glutamate receptor signaling pathway
	4.50E-02	10	87	Monosaccharide transmembrane transport
	4.50E-02	10	87	Hexose transmembrane transport
XtX and BF_{PC1} A	8.10E-04	5	23	Phloem development
	6.40E-03	29	1385	Macromolecule catabolic process
	1.00E-02	5	47	Phloem or xylem histogenesis
	1.30E-02	9	211	RNA phosphodiester bond hydrolysis, endonucleolytic
	1.30E-02	37	2140	Organic substance catabolic process
	1.60E-02	4	35	Salicylic acid metabolic process
	1.60E-02	35	2059	Cellular catabolic process
	1.60E-02	5	60	Protein complex oligomerization
	2.00E-02	4	40	Jasmonic acid metabolic process
	2.00E-02	4	41	Phenol-containing compound metabolic process
	2.00E-02	5	69	Response to hydrogen peroxide
	2.00E-02	7	155	RNA catabolic process
	2.00E-02	39	2450	Catabolic process
	2.00E-02	7	157	Response to salt stress
	2.40E-02	7	164	Response to osmotic stress
	2.70E-02	9	274	Polysaccharide catabolic process
	3.00E-02	9	282	RNA phosphodiester bond hydrolysis
	3.00E-02	6	127	Response to reactive oxygen species
	3.10E-02	15	670	Polysaccharide metabolic process
	4.90E-02	9	308	Organic hydroxy compound metabolic process
XtX and BF_{PC2} A	2.00E-03	5	36	Ionotropic glutamate receptor signaling pathway
	2.00E-03	5	36	Glutamate receptor signaling pathway
	7.30E-03	6	87	Monosaccharide transmembrane transport
	7.30E-03	6	87	Hexose transmembrane transport
XtX B	2.10E-05	13	48	Systemic acquired resistance
	2.10E-05	13	50	Defense response, incompatible interaction
	8.50E-03	15	115	Immune system process
	8.50E-03	15	113	Innate immune response
	8.50E-03	15	113	Immune response
	3.00E-02	8	41	Galactose metabolic process
BF_{PC1} B	3.00E-04	6	40	Jasmonic acid metabolic process
	3.00E-04	6	35	Salicylic acid metabolic process
	3.00E-04	6	41	Phenol-containing compound metabolic process
	2.90E-03	6	63	Benzene-containing compound metabolic process
BF_{PC2} B	1.60E-02	114	2450	Catabolic process
XtX and BF_{PC1} B	3.00E-02	3	40	Jasmonic acid metabolic process
	3.00E-02	3	35	Salicylic acid metabolic process
	3.00E-02	3	41	Phenol-containing compound metabolic process
	4.50E-02	31	4978	Protein modification process
	4.50E-02	3	63	Benzene-containing compound metabolic process
	4.50E-02	2	15	Chloroplast rRNA processing
	4.50E-02	31	4978	Cellular protein modification process
XtX and BF_{PC2} B	1.10E-02	62	2140	Organic substance catabolic process
	1.10E-02	68	2450	Catabolic process
	1.60E-02	6	41	Galactose metabolic process

Table S 2: Proportions of selection signature peak markers and of random markers with interchromosomal LD that surpasses LD thresholds. Only interchromosomal LD estimates were considered. Peak marker refers to the SNP with the local maximum in the differentiation or association statistic in the respective selection signal.

Scenario	LD threshold	All Signals	XtX	BF_{PC1}	BF_{PC2}	Random
A	LD > 0.21	0.138629205	0.184688471	0.254898803	0.100837277	2.82E-02
A	LD > 0.63	0.001706657	0.004281441	0.001878993	0.0001456134	4.45E-05
B	LD > 0.21	0.0504926335	0.108799295	0.03711448	0.0413032055	0.0283923241
B	LD > 0.63	0.0009526912	0.001865575	0.001568217	0.0009458749	0.0001759548

2 Supplementary Figures

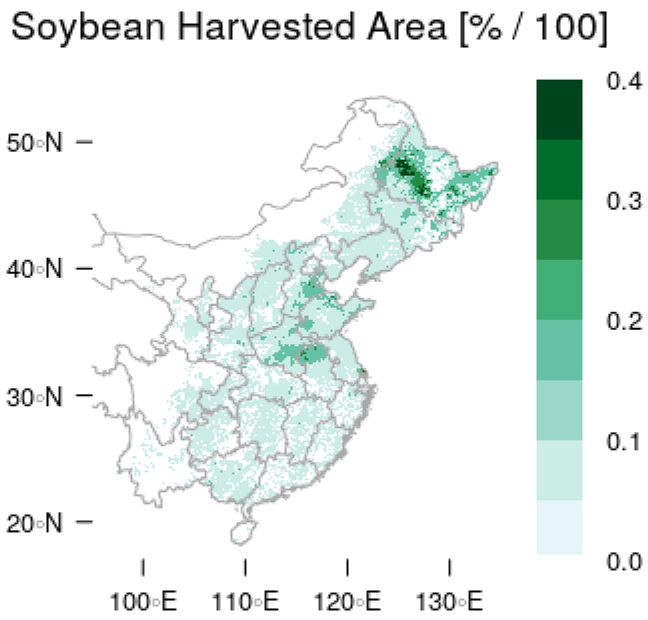

Figure S 1: Geographic extent of soybean cultivation in China (Ray et al. 2012).

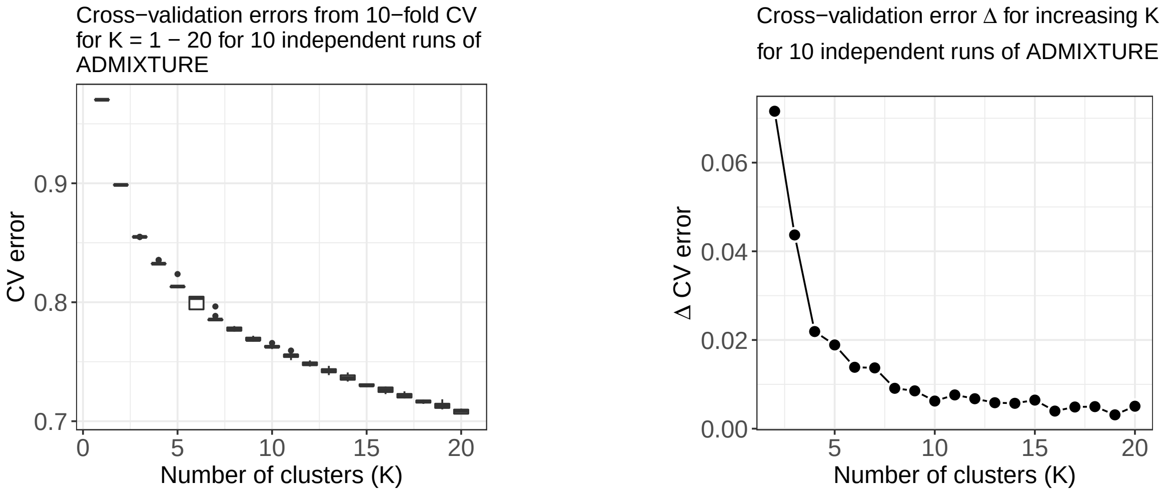

Figure S 2: Cross-validation error in the ADMIXTURE analysis for K varying from 1-20.

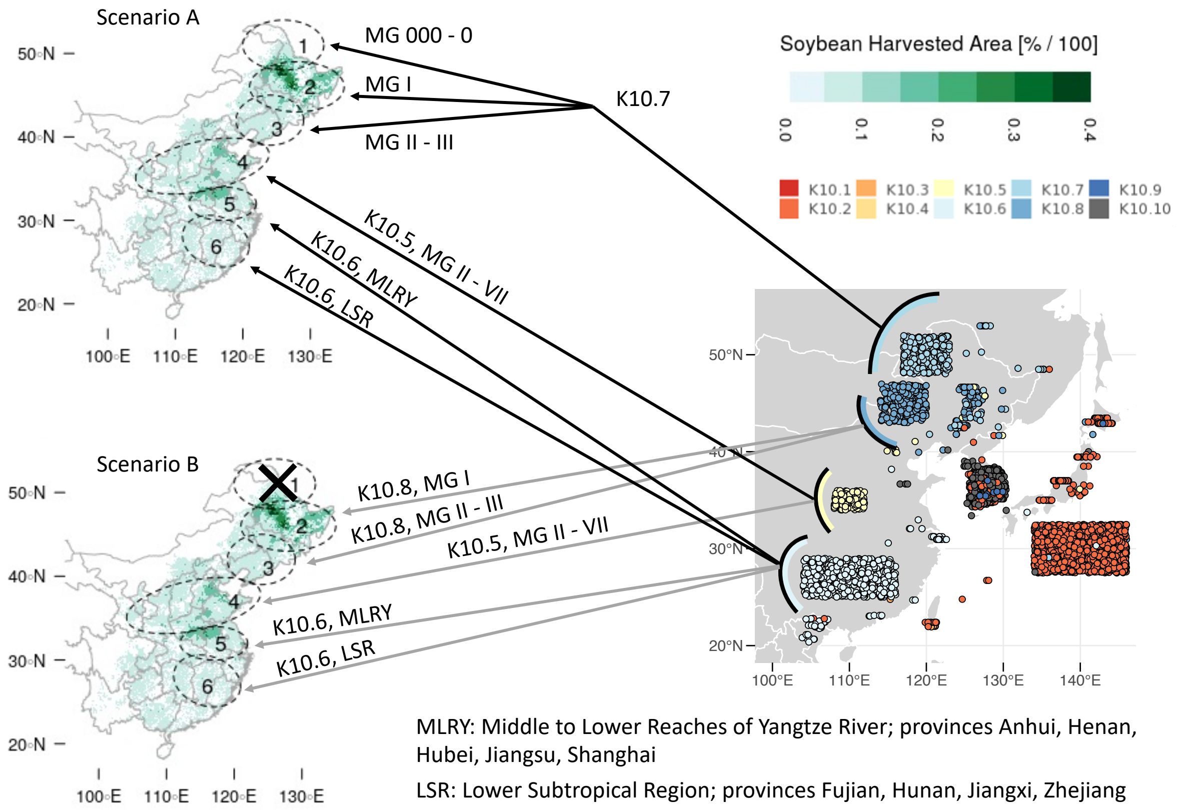

Figure S 3: Assignment of soybean germplasm groups with origin from China to growing regions based on population structure (ancestry >= 80%), maturity group ratings and provincial origin. 35% of accessions of group K10.7 (N = 525) have no recorded provincial origin, 26% are from "Northeast China", 14% from the North-Eastern Chinese provinces Heilongjiang, Jilin and Liaoning, and 11% from the Russian province Primorye. Accessions of group K10.8 (N = 379) are recorded to come from "Northeast China" in 23% of cases, 21% have no known provincial origin, 20% are from Heilongjiang, 22% from Jilin and 5% from Liaoning. K10.7 and K10.8 were subdivided according to maturity group ratings into the MG 000-0, MG I, and MG II-III subgroups which were assigned to growing regions 1-3, approximating their latitudinal adaptation. Accessions of group K10.5 (N = 269) in 32% of cases come from the Yellow River valley (Provinces Gansu, Heibei, Henan, Shaanxi, Shandong, Shanxi, Beijing) and 26% have no known provincial origin. We assigned 245 K10.5 accessions of MG II-VIII to growing region 4, covering the Yellow River valley. Accessions of the group K10.6 (N = 705) were assigned to growing region 5 (Middle to Lower Reaches of Yangtze River, N = 318) in cases with provincial origin from Anhui, Henan, Hubei, Jiangsu and Shanghai; and to growing region 6 (Lower Subtropical Region, N = 52) in cases with origin from Fujian, Hunan, Jiangxi and Zhejiang. Ancestry proportions, country-, and province level of origin are available in worksheet 1 of the extended supplementary for 18,020 genotypes.

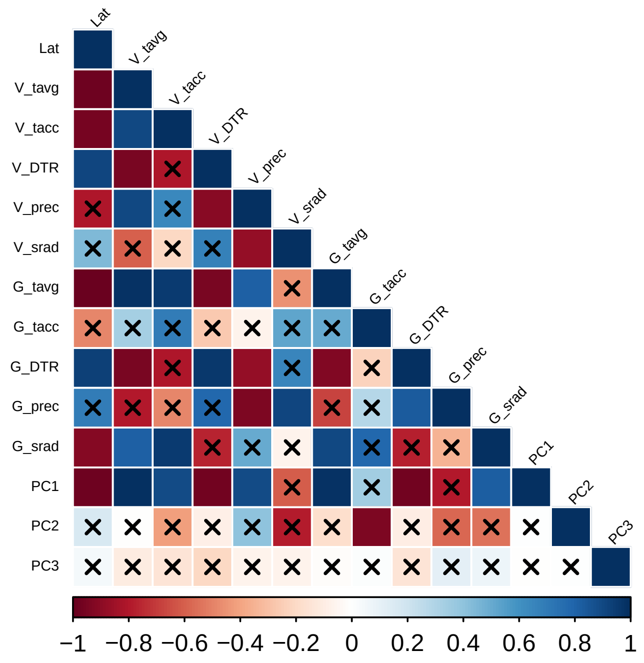

Figure S 4: Pearson correlation coefficients among environmental parameters characterizing six soybean growing regions in China. Black crosses indicate correlations that are not significant ($p > 0.05$).

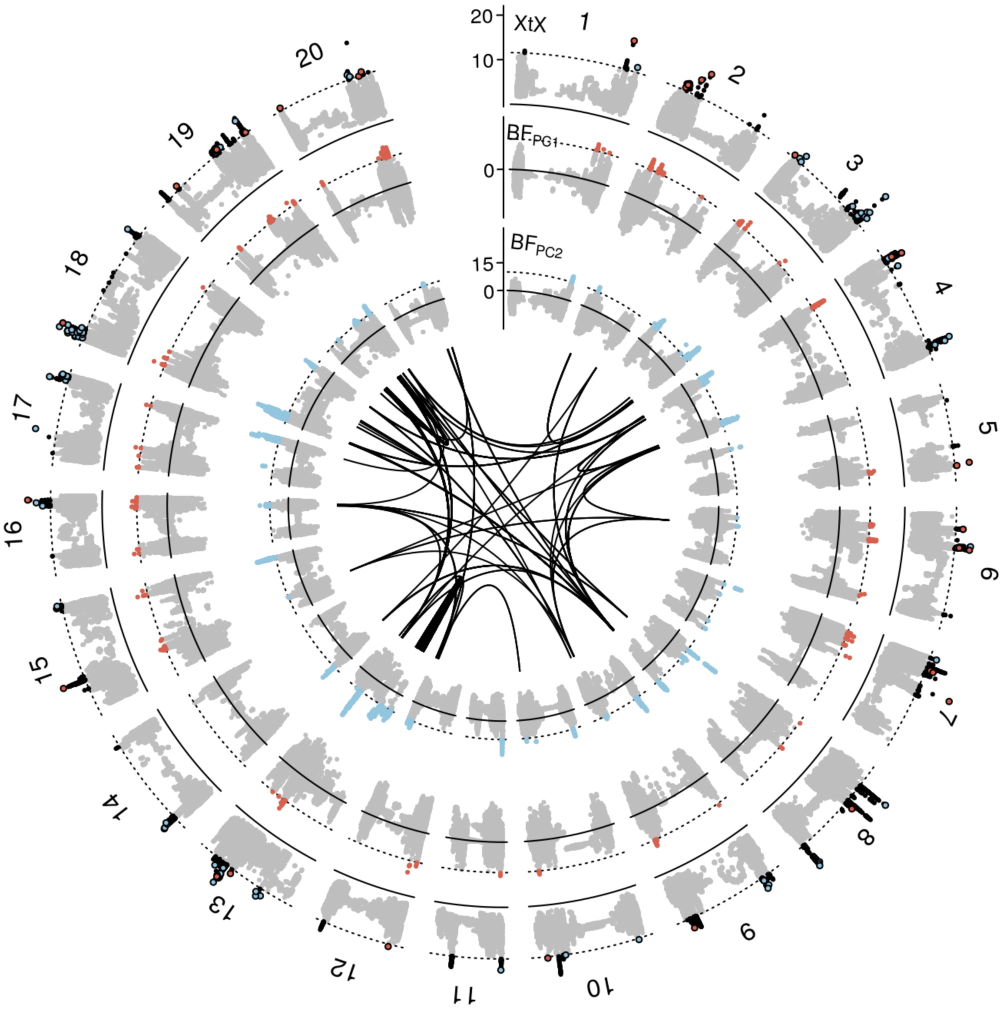

Figure S 5: Manhattan plot of the *XtX* statistic and of genotype-environment associations with the first two environmental principal components in Bayes factors for all 20 chromosomes in scenario B . Red and blue points in the *XtX* track represent overlaps between genetic differentiation and association signals with the first and second environmental principal components. The dotted horizontal lines represent the 1% POD significance threshold of the *XtX* statistic and the threshold of BF = 10 deciban. Black lines in the center represent regions with elevated LD exceeding the level of background LD 3-fold and a minimum physical distance of 5Mb between selection signatures.

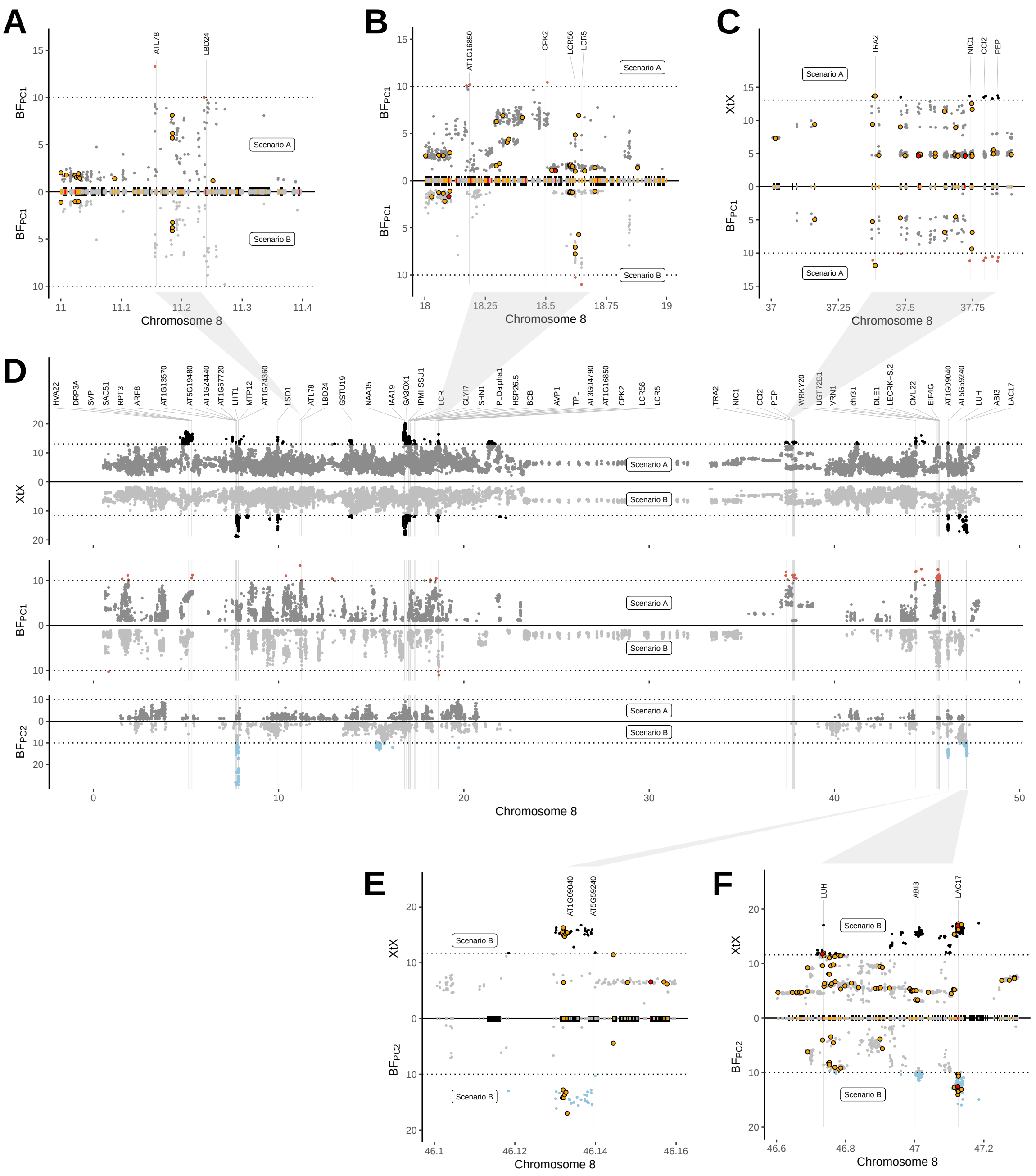

Figure S 6: Identification of candidate genes with a putative role in environmental adaptation on chromosome 8. (D) Manhattan plot of the *XtX* statistic and of genotype-environment associations with the first two environmental principal components in Bayes factors for scenario A and scenario B. Negative Bayes factors are omitted and dotted horizontal lines are analogous to Figure 2A. Labeled candidate genes are listed in Tab 3. Unit of x-axis is Mb. (A-C,E-F) Close-ups of genomic regions with selection signatures. Black blocks on the x-axis indicate the positions of predicted gene models. Red and yellow blocks and points indicate non-synonymous variants with high and moderate impacts on protein function.

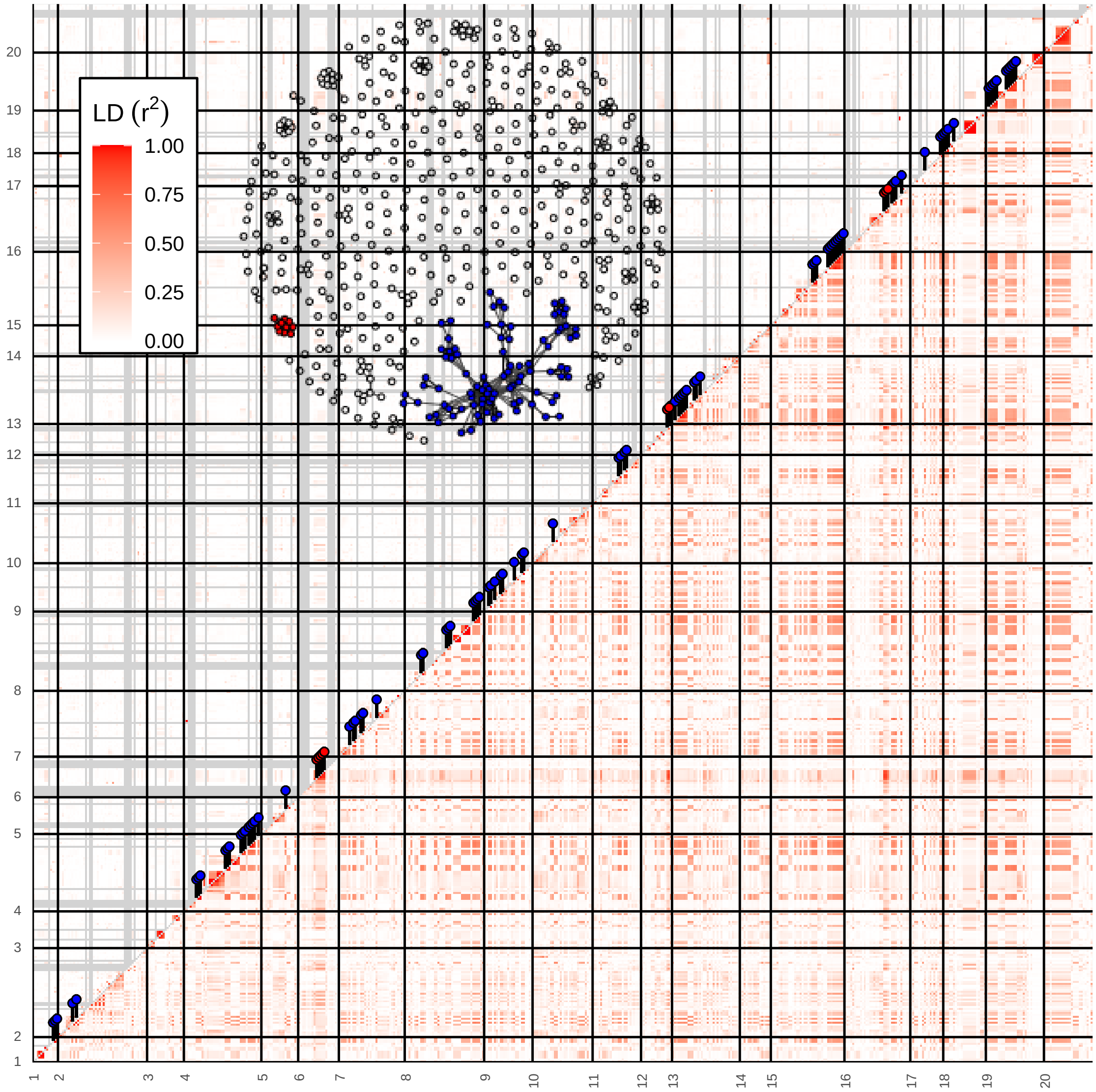

Figure S 7: LD estimates among 544 genomic regions with selection signatures observed in scenario A for germplasm groups from China (below diagonal) and in modern European soybean varieties (above diagonal). Red and blue pins on the diagnonal indicate regions that clustered in LD network analysis. Inset shows the LD network of the 544 genomic regions. Two clusters (92 and 10 regions, respectively) comprising LD connections exceeding the level of background LD 3-fold and a minimum physical distance of 5Mb are highlighted in blue and red.

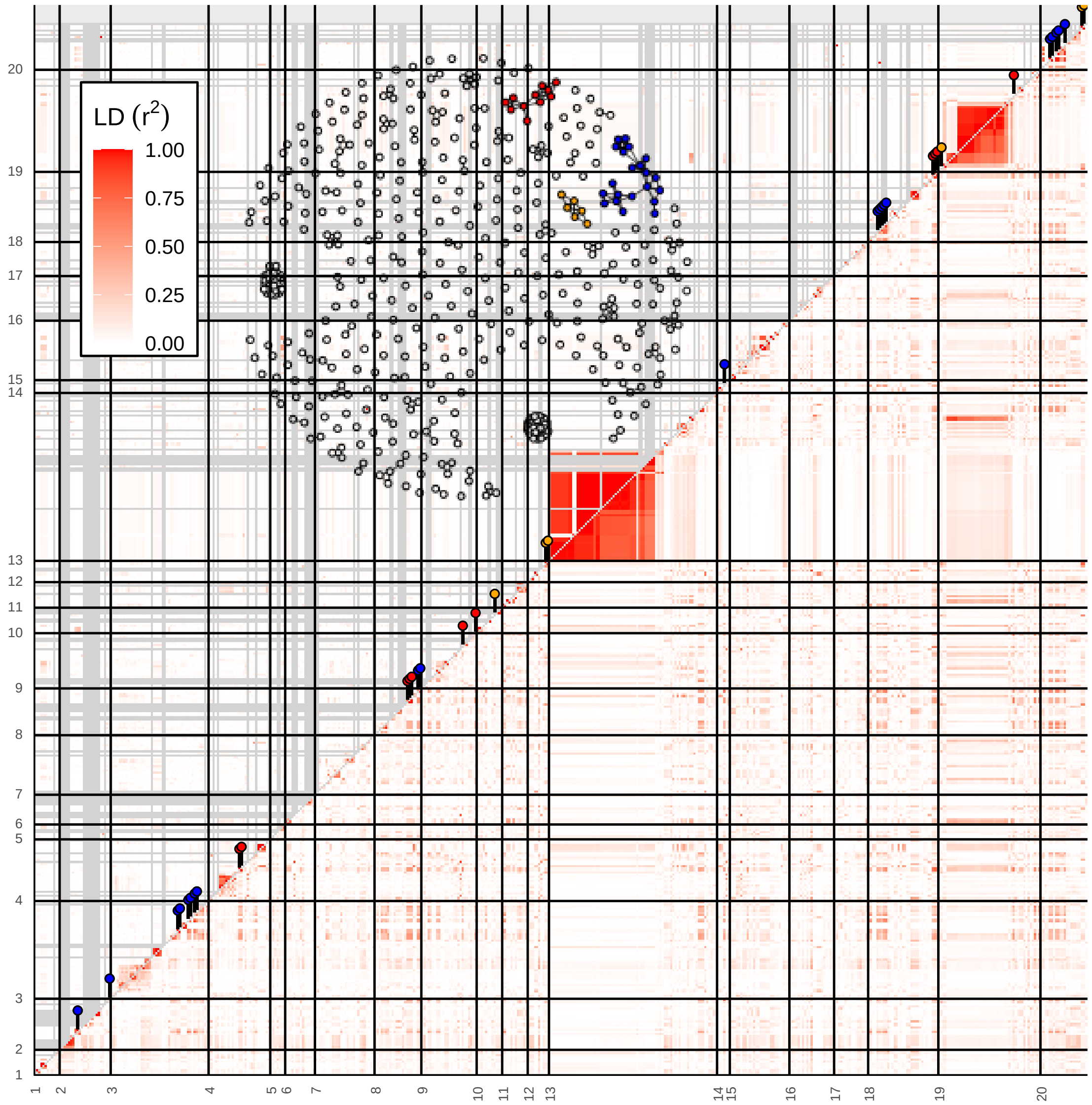

Figure S 8: LD estimates among 481 genomic regions with selection signatures observed in scenario B for germplasm groups from China (below diagonal) and in modern European soybean varieties (above diagonal). Red and blue pins on the diagnonal indicate regions that clustered in LD network analysis. Inset shows the LD network of the 481 genomic regions. Three clusters (21, 11 and 6 regions, respectively) comprising LD connections exceeding the level of background LD 3-fold and a minimum physical distance of 5Mb are highlighted in blue, red and orange.

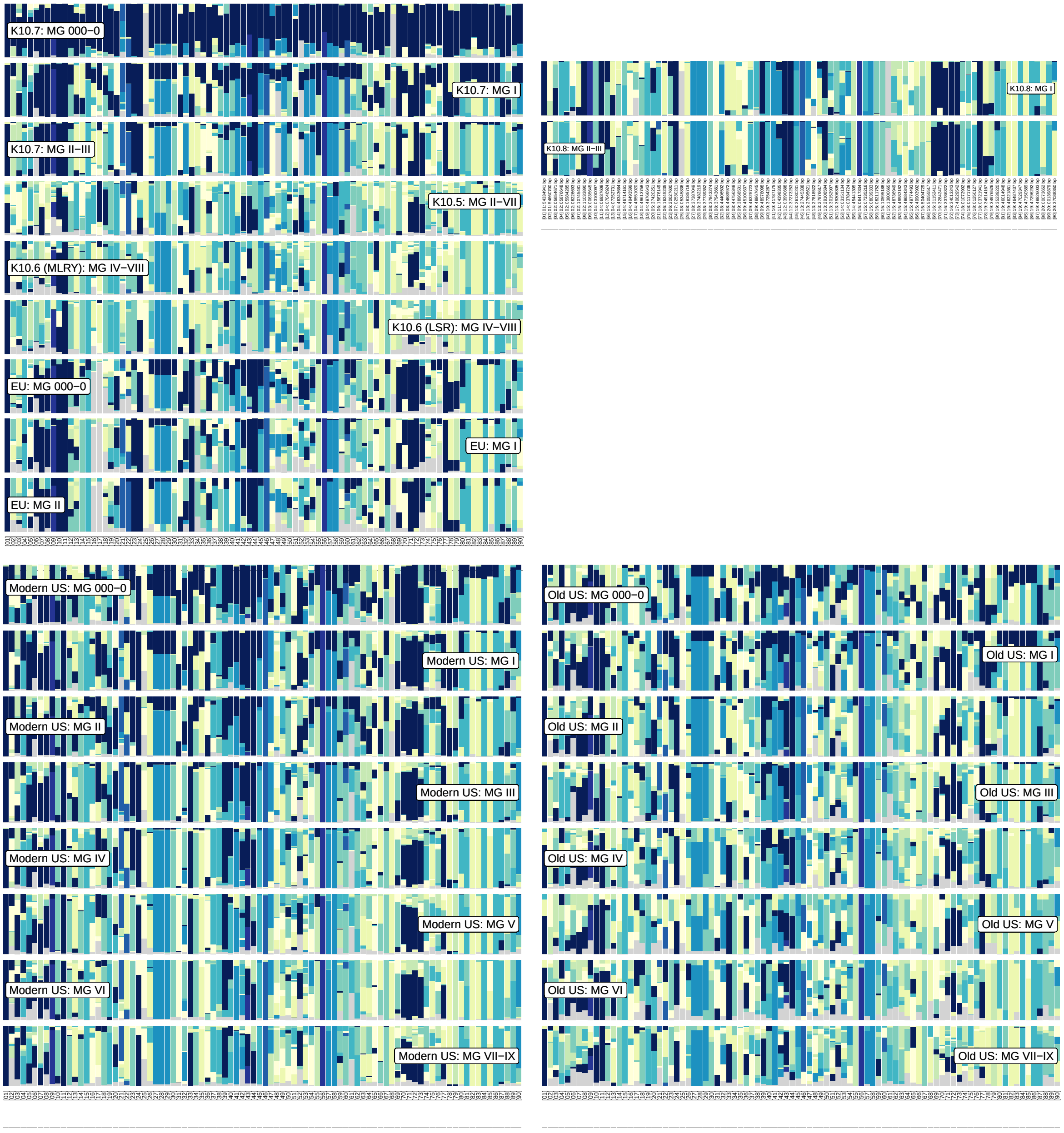

Figure S 9: Top left: Haplotype block proportions in germplasm groups from China (and modern European varieties) for 90 genomic regions with overlapping genetic differentiation and genotype-environment association signatures (PC1) observed in scenario A. Top right: Haplotype block proportions in the K10.8 subpopulation for the 90 genomic regions. Bottom left: Haplotype block proportions in modern US varieties for the 90 genomic regions. Bottom right: Haplotype block proportions in old US varieties for the 90 genomic regions.

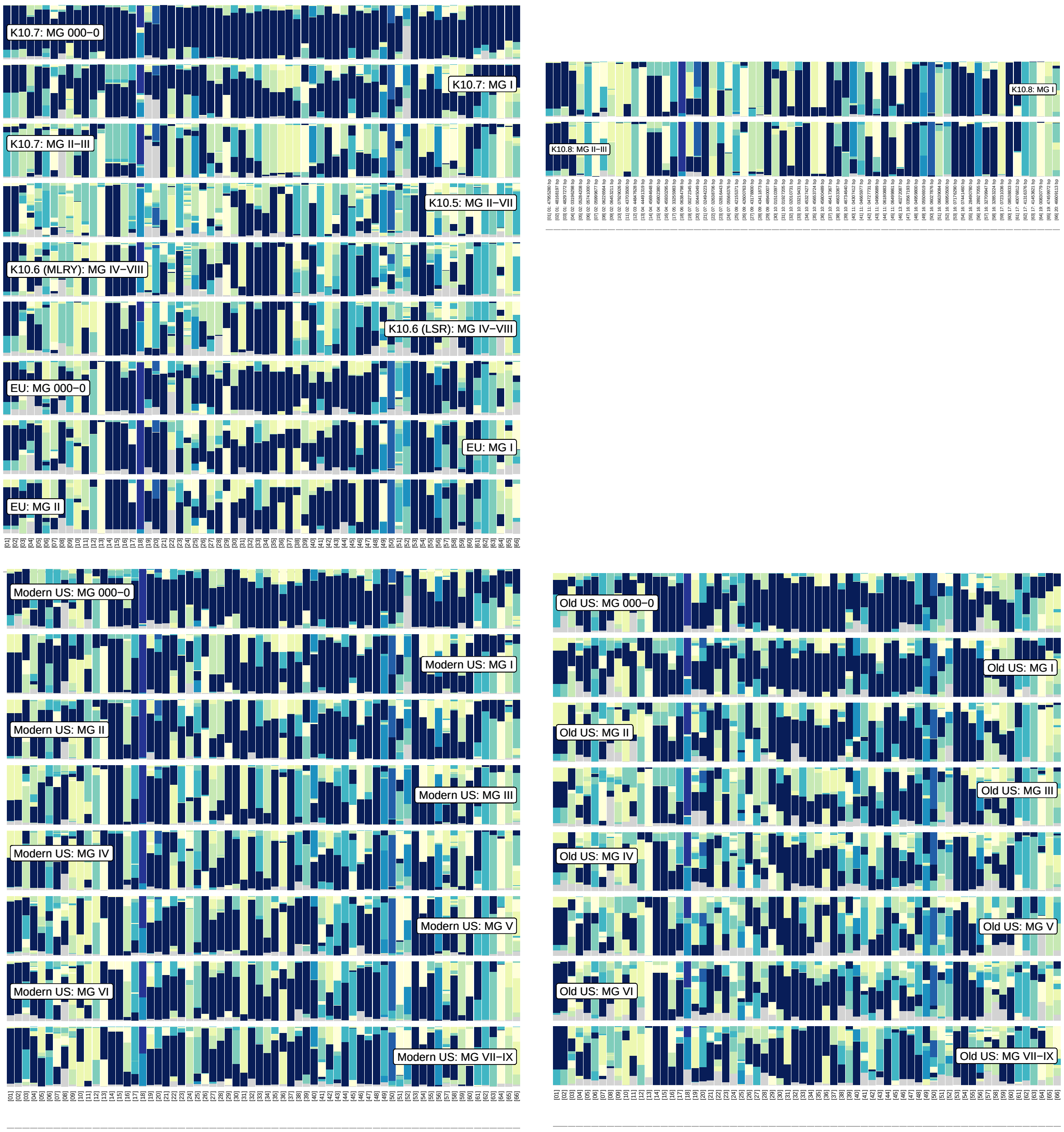

Figure S 10: Top left: Haplotype block proportions in germplasm groups from China (and modern European varieties) for 66 genomic regions with overlapping genetic differentiation and genotype-environment association signatures (PC2) observed in scenario A. Top right: Haplotype block proportions in the K10.8 subpopulation for the 66 genomic regions. Bottom left: Haplotype block proportions in modern US varieties for the 66 genomic regions. Bottom right: Haplotype block proportions in old US varieties for the 66 genomic regions.

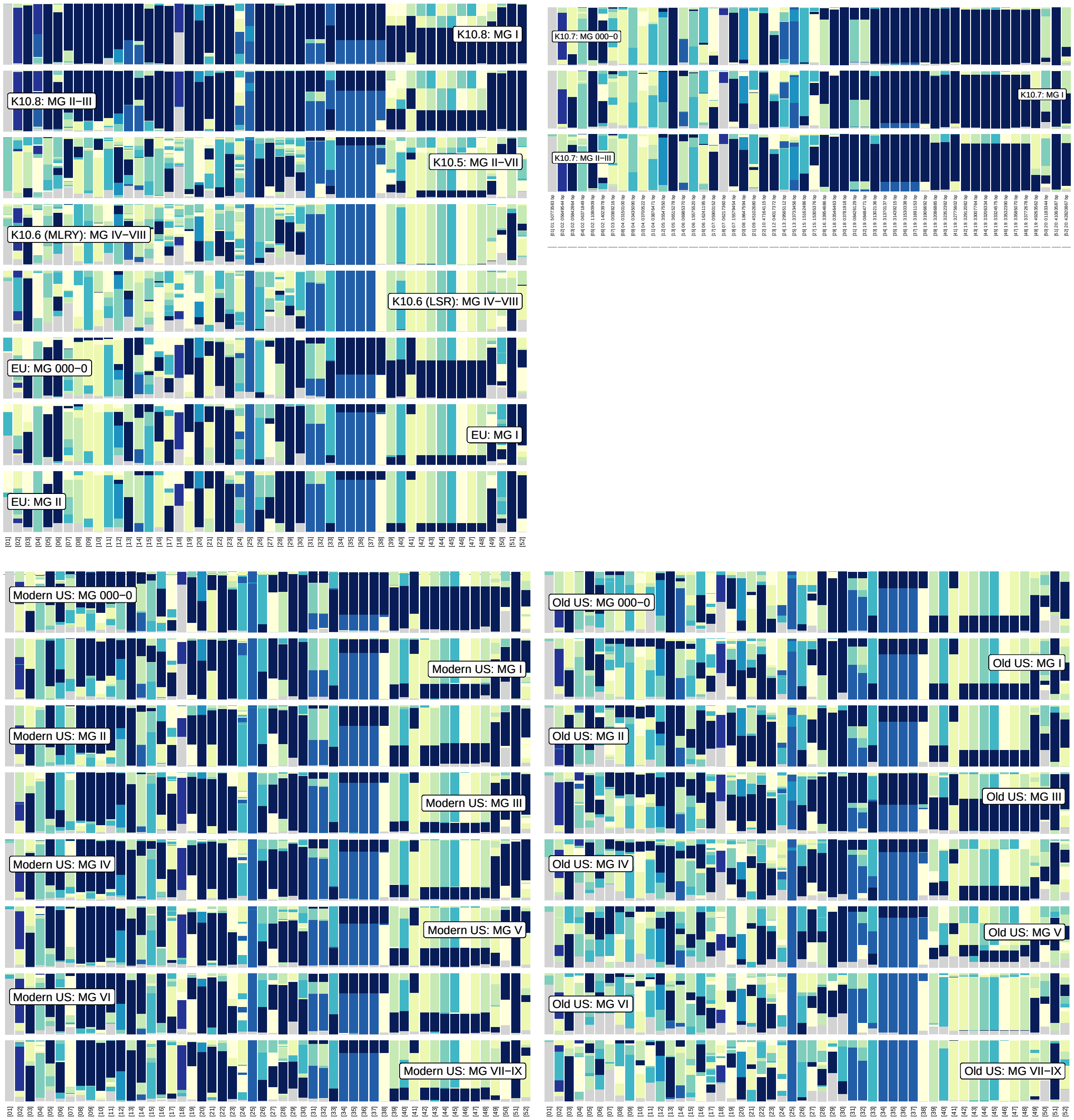

Figure S 11: Top left: Haplotype block proportions in germplasm groups from China (and modern European varieties) for 52 genomic regions with overlapping genetic differentiation and genotype-environment association signatures (PC1) observed in scenario B. Top right: Haplotype block proportions in the K10.7 subpopulation for the 52 genomic regions. Bottom left: Haplotype block proportions in modern US varieties for the 52 genomic regions. Bottom right: Haplotype block proportions in old US varieties for the 52 genomic regions.

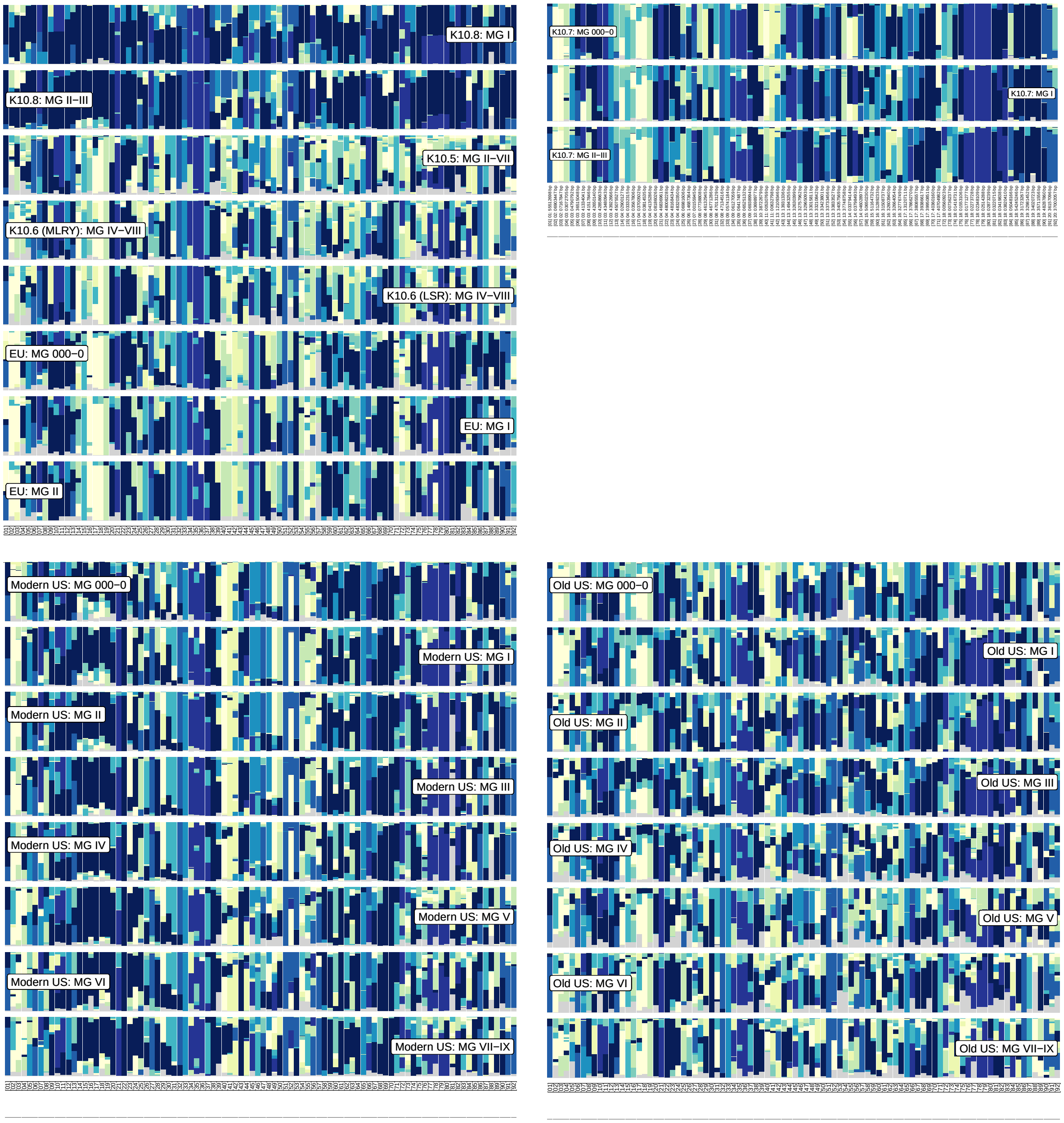

Figure S 12: Top left: Haplotype block proportions in germplasm groups from China (and modern European varieties) for 92 genomic regions with overlapping genetic differentiation and genotype-environment association signatures (PC2) observed in scenario B. Top right: Haplotype block proportions in the K10.8 subpopulation for the 92 genomic regions. Bottom left: Haplotype block proportions in modern US varieties for the 92 genomic regions. Bottom right: Haplotype block proportions in old US varieties for the 92 genomic regions.

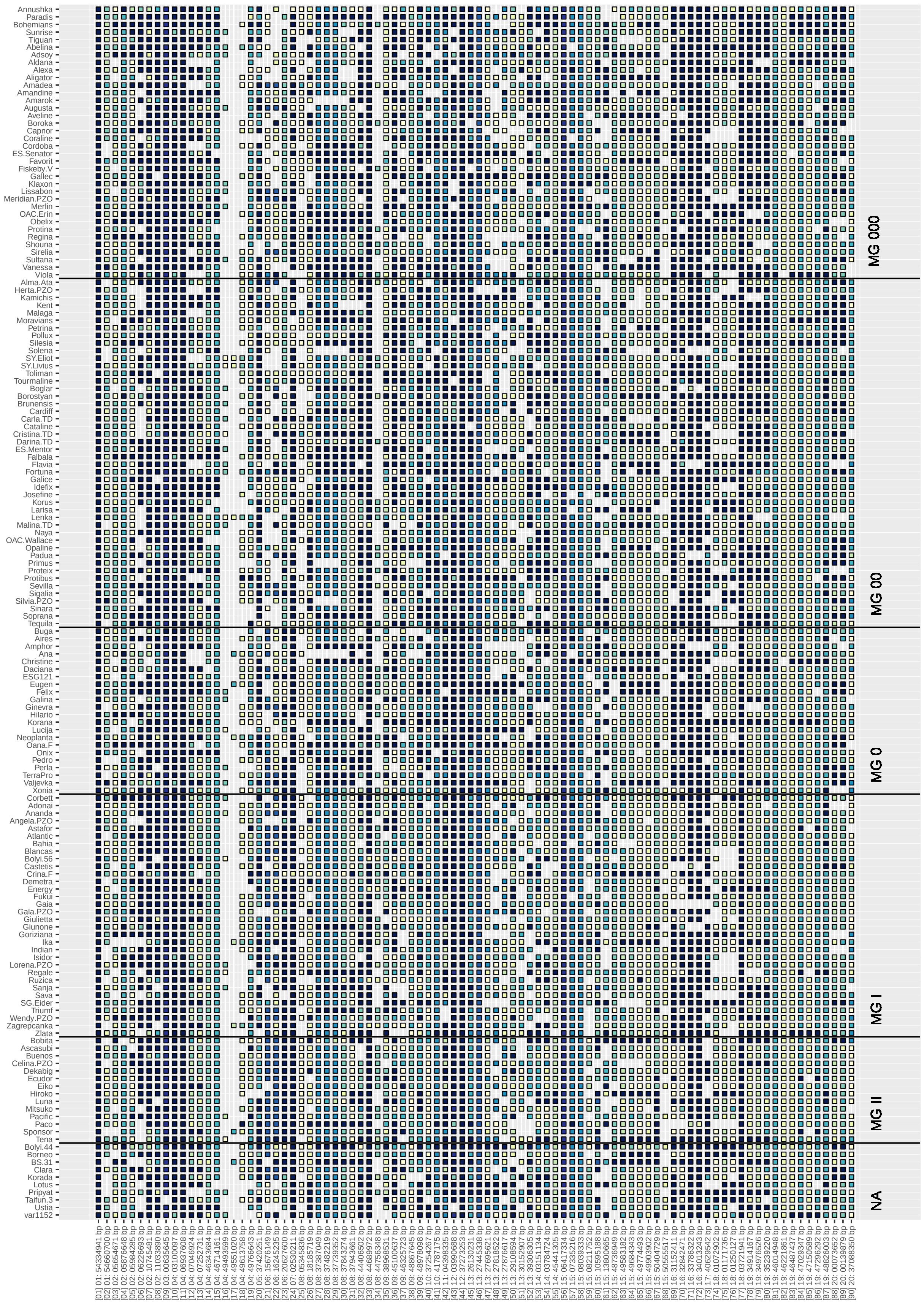

Figure S 13: Haplotype blocks in modern European varieties for 90 genomic regions with overlapping genetic differentiation and genotype-environment association signatures (PC1) observed in scenario A.

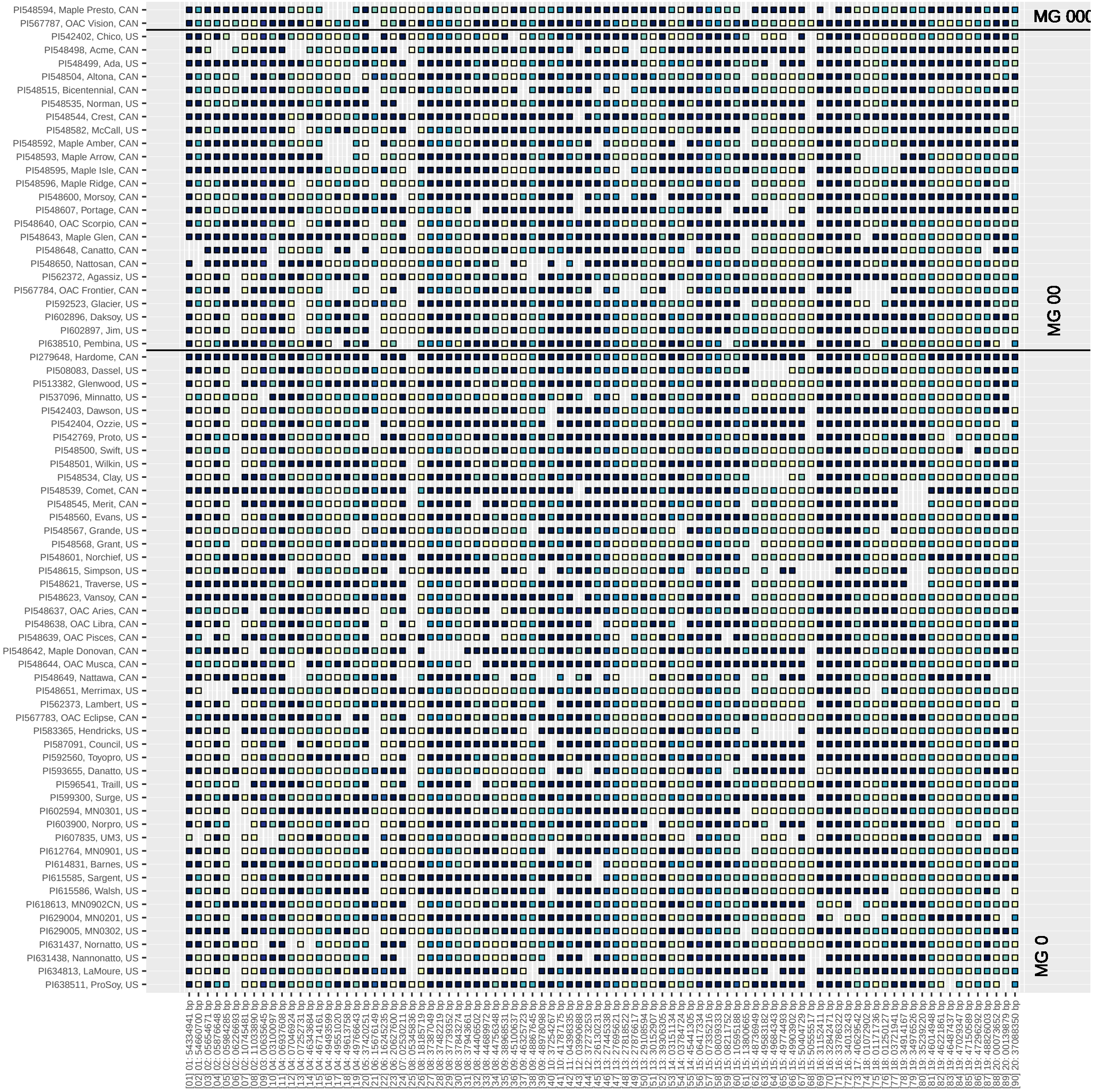

Figure S 14: Haplotype blocks in modern varieties from the USA and Canada for 90 genomic regions with overlapping genetic differentiation and genotype-environment association signatures (PC1) observed in scenario A.
